# Competition between mobile genetic elements drives optimization of a phage-encoded CRISPR-Cas system: insights from a natural arms race

**DOI:** 10.1098/rstb.2018.0089

**Published:** 2019-03-25

**Authors:** Amelia C. McKitterick, Kristen N. LeGault, Angus Angermeyer, Munirul Alam, Kimberley D. Seed

**Affiliations:** 1 Department of Plant and Microbial Biology, University of California, 111 Koshland Hall, Berkeley, CA 94720, USA; 2 International Centre for Diarrhoeal Disease Research, Dhaka, Bangladesh; 3 Chan Zuckerberg Biohub, San Francisco, CA 94158, USA

**Keywords:** CRISPR, phage, mobile genetic elements, cholera

## Abstract

CRISPR-Cas systems function as adaptive immune systems by acquiring nucleotide sequences called spacers that mediate sequence-specific defence against competitors. Uniquely, the phage ICP1 encodes a Type I-F CRISPR-Cas system that is deployed to target and overcome PLE, a mobile genetic element with anti-phage activity in *Vibrio cholerae*. Here, we exploit the arms race between ICP1 and PLE to examine spacer acquisition and interference under laboratory conditions to reconcile findings from wild populations. Natural ICP1 isolates encode multiple spacers directed against PLE, but we find that single spacers do not interfere equally with PLE mobilization. High-throughput sequencing to assay spacer acquisition reveals that ICP1 can also acquire spacers that target the *V. cholerae* chromosome. We find that targeting the *V. cholerae* chromosome proximal to PLE is sufficient to block PLE and is dependent on Cas2-3 helicase activity. We propose a model in which indirect chromosomal spacers are able to circumvent PLE by Cas2-3-mediated processive degradation of the *V. cholerae* chromosome before PLE mobilization. Generally, laboratory-acquired spacers are much more diverse than the subset of spacers maintained by ICP1 in nature, showing how evolutionary pressures can constrain CRISPR-Cas targeting in ways that are often not appreciated through *in vitro* analyses.

This article is part of a discussion meeting issue ‘The ecology and evolution of prokaryotic CRISPR-Cas adaptive immune systems’.

## 1. Introduction

Phages often vastly outnumber their bacterial hosts in a variety of environments [1]. As such, bacteria have evolved numerous mechanisms for phage defence, including adaptive immunity via clustered regularly interspaced short palindromic repeats (CRISPR) and CRISPR-associated (Cas) proteins [2,3]. CRISPR-Cas systems are composed of a CRISPR array—a series of ‘spacers’ of foreign sequence alternating with repeats that are transcribed into CRISPR RNAs (crRNAs)—and *cas* genes. Together with crRNAs, Cas proteins defend against foreign nucleic acids, such as the genome of an infecting phage, through a three-step process: adaptation, crRNA and *cas* gene expression, and interference. During adaptation, a foreign DNA fragment is incorporated into the CRISPR array to provide a molecular memory of the challenges that the host cell has faced. This CRISPR array is expressed and processed into individual crRNAs, which complex with Cas proteins and survey the cell for complementary invading nucleotides. Upon finding a complementary sequence, termed a protospacer, a Cas nuclease is recruited to the site to mediate interference by cleaving the substrate, ultimately leading to the destruction of the invader [3,4]. Across CRISPR-Cas containing bacteria and archaea, Class 1 Type I CRISPR-Cas systems employing a Cas3 enzyme for DNA unwinding and degradation [5] are the most prevalent [6].

**Figure 1.**
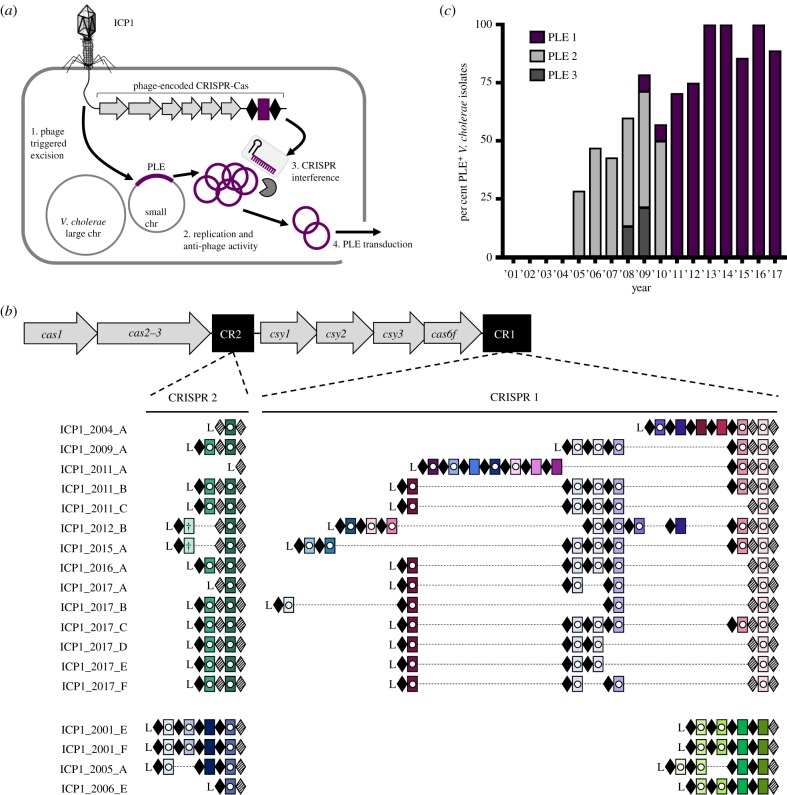
ICP1 uses CRISPR-Cas to overcome epidemic *V. cholerae* PLE. (*a*) Lytic phage ICP1 infects *V. cholerae* triggering PLE excision. PLE replicates and exerts anti-phage activity, ultimately leading to PLE transduction. Concurrently, ICP1-encoded CRISPR-Cas is expressed to interfere with PLE activity. (*b*) The architecture of the ICP1 CRISPR-Cas system and comparison of spacer composition between phage isolates. For each CRISPR locus, the repeat (28 bp) and spacer (32 bp) content is detailed as black diamonds and coloured rectangles, respectively. Repeats that match the repeat consensus [13] are shown by solid diamonds, and degenerate repeats are indicated by hatched black diamonds. An AT-rich leader sequence (L) precedes each CRISPR locus. Identical spacers shared between isolates are shown as rectangles with identical colours. Spacers containing a white circle target PLE, and spacers containing a cross target the *V. cholerae* large chromosome. (*c*) Percentage of *V. cholerae* isolates harbouring PLE recovered from epidemic sampling at the International Centre for Diarrhoeal Disease Research, Bangladesh over time (*n* = 230 strains analysed).

CRISPR-Cas systems do not discriminate between horizontally acquired traits based on fitness gain or loss. Hence, CRISPR-Cas systems are equally capable of halting harmful invading phage DNA as they are halting beneficial mobile genetic elements, including those encoding antibiotic resistance and pathogenicity genes [7–9]. As such, some pathogens only have alternative anti-phage defence systems [10]. For example, the currently circulating biotype of epidemic *Vibrio cholerae*, the causative agent of the diarrheal disease cholera, does not rely on CRISPR-Cas for phage defence [11]. Instead, *V. cholerae* evolved to use phage inducible chromosomal island-like elements (PLEs) to defend against the prevalent lytic phage, ICP1 [12]. PLEs are mobile genetic elements that reside integrated in the small chromosome of *V. cholerae* [12]. During ICP1 infection of PLE(+) *V. cholerae*, PLE excises from the host chromosome, replicates to high copy and is horizontally transduced to naive neighbouring cells, all the while inhibiting phage replication through unknown mechanisms (figure 1*a*).

In order to overcome the anti-phage activity encoded by *V. cholerae* PLE, some ICP1 isolates use a Type I-F CRISPR-Cas system that directly targets PLE (figure 1*a*), making the CRISPR-Cas system essential for the phage to form plaques on PLE(+) *V. cholerae* [13]. Type I-F systems are composed of three Csy proteins that make up the Csy complex along with Cas6f, a protein involved in crRNA processing [14]. This complex interacts with the processed crRNA to search DNA for a complementary protospacer with an appropriate self versus non-self discrimination sequence, known as the protospacer adjacent motif (PAM) [15]. Upon finding a match with an appropriate PAM, the trans-acting Cas2-3 fusion protein is recruited to degrade the target DNA. In addition to endonuclease activity, Cas2-3 has a helicase domain that unwinds DNA as the protein translocates away from the target DNA, allowing for continued processive degradation of adjacent DNA *in vitro* [16,17]. Recently, sequence analysis identified phages that are predicted to encode CRISPR arrays and/or *cas* genes [18,19]; however, ICP1 is the only phage shown to encode a fully functional CRISPR-Cas system [12,13].

As is true when CRISPR-Cas is harnessed by a prokaryotic host for genome defence, the ICP1-encoded CRISPR-Cas system is tasked with targeting and degrading a hostile mobile genetic element. However, there are additional challenges associated with a phage encoding and relying on CRISPR-Cas for its own survival. The ICP1 infection cycle occurs over a 20 min period, and current data suggest that ICP1 synthesizes its CRISPR-Cas machinery de novo upon infection of *V. cholerae* [13]. PLE is induced to excise within minutes of infection through interactions with an early phage-encoded gene product [20]. Thus, in order to overcome PLE, CRISPR synthesis and interference must outpace a rapidly replicating target.

ICP1 and *V. cholerae* are consistently co-isolated from patient stool samples in regions where cholera is endemic, such as Bangladesh [12,21,22]. Five genetically distinct PLE variants in *V. cholerae* have appeared in temporally discrete waves across cholera epidemics [12]. Previous analysis revealed that ICP1-encoded CRISPR-Cas can adapt and acquire new spacers against PLE under laboratory conditions [13], however the rules governing spacer acquisition and targeting efficacy for this system are not known. Further, recent comparative genomics of 18 ICP1 isolates collected from Bangladesh between 2001 and 2012 found that 50% carry CRISPR-Cas [23], however the contemporary state of circulating ICP1 and *V. cholerae* PLE in the region are not known.

Here, we provide an up-to-date understanding of the genomic variants of ICP1 and PLE circulating in Bangladesh. We find that natural ICP1 isolates encode multiple anti-PLE spacers and experimentally validate that increased PLE targeting by ICP1 is required to fully abolish PLE mobilization. Significantly, using a high-throughput spacer acquisition assay and experimental validation, we show that non-canonical PAMs and indirect protospacers in the *V. cholerae* small chromosome can unexpectedly provide protection against PLE. Our results support a model in which ICP1-encoded CRISPR-Cas that is directed against the *V. cholerae* small chromosome is in a race to reach PLE before it excises from the chromosome to exert its anti-phage activity. Taken together, our study highlights the differences between interference competent spacers under laboratory conditions and those that are selected for in nature to provide mechanistic insight into the evolutionary pressures governing the interactions between epidemic *V. cholerae* and its longstanding battle with the predatory phage ICP1.

## 2. Methods

### (a) Strains, growth conditions and genomic analysis

Phage, bacterial strains and plasmids used in this study are listed in electronic supplementary material, tables S1–S3. Bacteria were routinely grown at 37°C on lysogeny broth (LB) agar or in LB broth with aeration. Media was supplemented with ampicillin (50 µg ml^−1^), kanamycin (75 µg ml^−1^), spectinomycin (100 µg ml^−1^) and/or streptomycin (100 µg ml^−1^) when appropriate. Phage susceptibility was determined by standard soft agar overlays as described [11] and phage plaque spot plates were performed as described previously [20]. Images are representative of at least two independent assays. Cholera stool samples collected and stored at the ICDDR,B between 2015 and 2017 were probed for the presence of phage by standard soft agar overlays, and *V. cholerae* isolates were recovered by plating on Thiosulfate Citrate Bile Salts Sucrose selective media (Difco). ICP1-specific primers [13,22] and PLE-specific primers (electronic supplementary material, table S4) were used for preliminary screening of isolates from stool samples. The presence of CRISPR-Cas in ICP1 and PLE in *V. cholerae* was validated by whole-genome sequencing. Genomic libraries were generated using NEBNext Ultra II DNA Library preparation kit for Illumina (New England Biolabs), according to the manufacturer's recommended protocols. Paired-end sequencing (2 × 150 bp) was performed on an Illumina HiSeq4000 (University of California, Berkeley QB3 Core Facility). Sequencing assembly/mapping and detection of CRISPR was performed as described [23]. The genome of the *V. cholerae* clinical isolate KS393 was sequenced on Illumina HiSeq4000, PacBio Sequel and Oxford Nanopore MinION sequencers (University of California, Berkeley QB3 Core Facility). Assembly of KS393 sequences was performed using the canu assembler v1.6 [24] to combine the PacBio and Oxford Nanopore reads into genomic scaffolds for the large and small chromosomes using default settings and an expected genome size of 4033460 bp. This generated two scaffolds of the expected sizes for each chromosome which were then polished with the Illumina paired-end sequences using Pilon v1.22 [25] with the ‘fix all’ command to generate a high-quality genomic assembly in a fasta format of both chromosomes (electronic supplementary material, File S1). The sequencing data for strain KS393 have been deposited in the Sequence Read Archive (SRA) database under accession codes SRR7826356, SRR7826357 and SRR7826358.

*Vibrio cholerae* mutants were constructed by natural transformation as described [26]. Mutations in ICP1 were generated using CRISPR-Cas mediated genome engineering with the *V. cholerae* classical biotype Type I-E system as described [11] (electronic supplementary material, tables S3 and S4). Engineered phage ± Cas1 D244A with spacer 9 were validated by plaquing on a permissive PLE 1 host and determining the frequency of phage with a newly acquired spacer by calculating the efficiency of plaquing on the permissive PLE 1 host to a PLE 1 host with the protospacer deleted (PLE 1^ΔPS9^). The limit of detection is met when the phage is unable to form a plaque on the restrictive host at the highest concentration while still being able to productively infect a permissive host, with at least 6 orders of magnitude tested. Examination of PLE replication and transduction during phage infection was as reported previously [12].

### (b) High-throughput spacer acquisition, data processing and analyses

Three independent experiments were performed as follows: a 50 ml culture of PLE 1 *V. cholerae* was grown to OD_600_ = 0.3 and infected at a multiplicity of infection of 1 with ICP1_2011_A ΔS9, which harbours spacer 8 at the leading edge of the CRISPR 1 array that targets PLE 1 and allows for phage replication [13]. Infected cells were incubated for 90 min at 37°C with aeration, at which point lysis was observed. The lysate was treated with chloroform and centrifuged to remove bacterial debris. Phage were precipitated with 10% (w/v) polyethylene glycol (PEG) 8000 at 4°C overnight. Phage pellets were collected by centrifugation at 4°C and the passaging was repeated as above. After three passages, the resulting pools were plated on a host possessing silent mutations in protospacer 8 (PLE 1^PS8^^*^) that inhibits spacer 8-mediated CRISPR interference, which enabled the selection of phage with expanded arrays that allow plaque formation. Phage DNA libraries were generated by homopolymer tail-mediated PCR (HTM-PCR) as previously described [27]. As ICP1_2011_A possesses only a single functional CRISPR array (figure 1*b*), the expanded phage CRISPR 1 array was amplified from genomic DNA libraries by PCR using custom barcoded primers (electronic supplementary material, table S4) to sequence the leader proximal spacer. 50 bp single-end sequencing was performed on an Illumina HiSeq 2500 (Tufts University Core Facility) using a custom sequencing primer. The resulting reads as fastq files were mapped to the large and small chromosome of *V. cholerae* strain KS393 using Bowtie v1.2.2 [28] with a seed_length of 31 and allowing for 0 max_total_mismatches which ensured that spacer to protospacer matches were 100% identical. These mappings were performed in two parallel ways: first, to obtain all possible spacer mapping locations regardless of the number of identical protospacer targets (i.e. translucent spacers in figure 3*b*) and second, restricting max_alignments to 1 which only mapped spacers with exactly one unique mapping location across both chromosomes. With a custom Python script (https://git.io/fNVqZ) we extracted the PAM sequences and GG PAM slippage locations from the restricted unique mappings. We also used this script to generate spacer mapping location graphs for both set of mappings using Biopython's GenomeDiagram module [29]. The amplicon sequencing data have been deposited in the SRA database under accession codes SRR7827053, SRR7827054 and SRR7827055.

## 3. Results

### (a) ICP1-encoded CRISPR-Cas is fixed in the natural phage population

We set out to compare ICP1 and PLE from contemporary cholera patient stool samples to previously identified isolates from the International Centre for Diarrhoeal Disease Research, in Dhaka, Bangladesh (ICDDR,B) [13,21]. We isolated eight new ICP1 isolates from cholera patient stool samples collected between 2015 and 2017 and found that all isolates harbour CRISPR-Cas. Thus, it appears that ICP1 isolates lacking CRISPR have not been identified in Bangladesh since 2006 [23]. Analysis of the CRISPR arrays indicates a strong selection for spacers specifically targeting PLE (figure 1*b*; electronic supplementary material, table S5), supporting the function of the ICP1-encoded CRISPR-Cas system as a counter-attack against the anti-phage island PLE [13]. To evaluate if the fixation of CRISPR in ICP1 is necessitated by co-circulating PLE in epidemic *V. cholerae*, we determined the prevalence of PLE over the same near two-decade long period in Dhaka. Combined with previous analyses [12,21], we observed an increase in the prevalence of PLE(+) *V. cholerae* in epidemic sampling over time (figure 1*c*). Of note is the high prevalence of PLE 1 *V. cholerae* over the past 6 years, indicating that this variant of the anti-phage island is currently dominating the epidemic landscape in Dhaka. Despite the relatively long period over which PLE 1 has been dominant in Dhaka, and consistent with previous results [12,21], whole-genome sequencing of eight PLE 1 *V. cholerae* isolates showed that PLE 1 is 100% identical at the nucleotide level in all strains.

### (b) Multiple spacers increase ICP1 CRISPR-Cas mediated PLE interference

**Figure 2.**
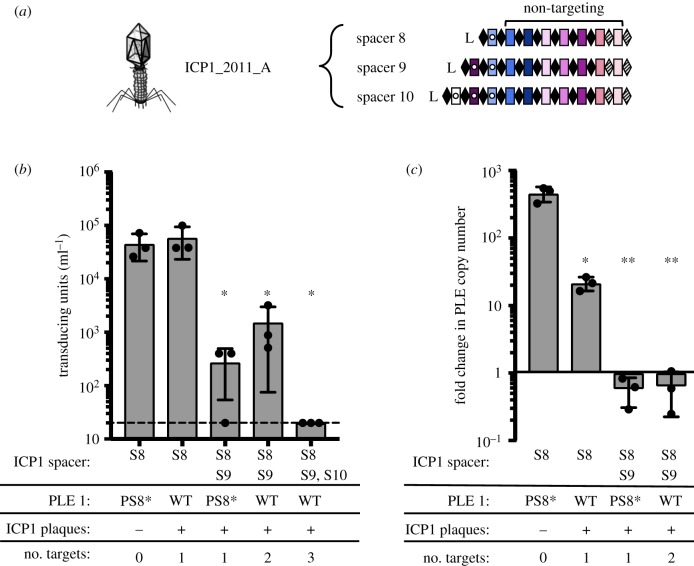
CRISPR can limit horizontal transmission of PLE. (*a*) ICP1_2011_A with anti-PLE 1 spacers S8, S9 and S10 (shown with internal white circles) tested in panels (*b*) and (*c*). (*b*) PLE transduction after infection with ICP1 with 0, 1, 2 or 3 spacers. The dashed line indicates the limit of detection for this assay. PS8* indicates silent mutations in protospacer 8 that abolish CRISPR interference [13]. A single spacer is necessary and sufficient to permit lytic growth of ICP1 on PLE 1 *V. cholerae* as seen by equal plaque formation (indicated by +, electronic supplementary material, figure S1). (*c*) PLE replication 20 min after infection with ICP1 with 0, 1 or 2 spacers as determined by qPCR. For panels (*b*,*c*), error bars indicate standard deviations of biological triplicates. Significance was determined by *T*-test, *p < 0.05, **p < 0.005.

All of the natural phage we isolated encode multiple CRISPR spacers against PLE (figure 1*b*); however, previous work revealed that only one functional spacer is required for ICP1 to overcome PLE-mediated anti-phage activity as evaluated by plaque formation (electronic supplementary material, figure S1) [13]. Conversely, a single spacer against the PLE did not prevent transduction of PLE [12]. To investigate the consequences of varying spacer number and identity on PLE transduction and replication, we used co-isolated ICP1 and PLE 1 *V. cholerae* obtained from a cholera patient sample in 2011 [13]. This ICP1 isolate harbours two spacers (spacers 8 and 9) at the leading edge of the CRISPR 1 array that target PLE 1. We also used an isogenic phage with a spontaneous loss of spacer 9 [13], as well as one that acquired an additional 10th spacer targeting PLE *in vitro* (figure 2*a*). Despite the ability to overcome PLE and form plaques, spacer 8 targeting was not sufficient to decrease PLE transduction during ICP1 infection relative to an untargeted control (figure 2*b*). In comparison, two anti-PLE spacers decreased PLE transduction during ICP1 infection and three spacers completely abolished PLE transduction, showing that increased CRISPR targeting by ICP1 has a stronger anti-PLE effect. To evaluate potential differences between spacer 8 and spacer 9 on PLE targeting, we used PLE 1 with a protospacer mutation (PLE 1^PS8*^) that inhibits spacer 8-mediated PLE targeting [13]. Strikingly, just spacer 9 targeting PLE alone was able to decrease PLE transduction to the same level as when two spacers were targeting PLE (figure 2*b*).

We next analysed the copy number of PLE during infection with ICP1 encoding one or two targeting spacers to identify if the differences in reducing PLE transduction were due to differences in PLE copy number (figure 2*c*). In the absence of ICP1 CRISPR targeting, PLE replicates to high copy number, which facilitates horizontal transmission [12]. Targeting with only one spacer was sufficient to significantly decrease PLE replication, and in agreement with the transduction data, spacer 9 had a stronger inhibitory effect on PLE replication than spacer 8. Sequencing of the newly transduced PLEs showed no mutations in the protospacers. Thus, the transduced PLEs did not escape CRISPR targeting through mutation, but instead, the individual spacers possess different and incomplete abilities to fully block PLE mobilization. Altogether, these results demonstrate that not all spacers selected in nature equally interfere with PLE mobilization and that increasing the number of spacers provides enhanced capacity of ICP1 to interfere with PLE.

### (c) Interference-driven spacer acquisition in ICP1 reveals indirect targets and non-canonical protospacer adjacent motifs

Since spacer composition variability in nature was lower than we expected (figure 1*b*), we next set out to experimentally sample the repertoire of spacers that ICP1 can acquire to overcome PLE. Low-throughput experiments previously demonstrated that ICP1 can acquire new spacers targeting the PLE under laboratory conditions without the need to overexpress *cas* genes [13]. To further analyse the natural process of interference-driven spacer acquisition in this system, we performed high-throughput sequencing of expanded CRISPR arrays of phage selected on PLE 1 *V. cholerae*. We infected PLE 1 *V. cholerae* with ICP1 containing spacer 8 (figure 2*a*), and the recovered lysate was probed for ICP1 progeny with newly acquired spacers that allowed for plaque formation on a PLE 1^PS8*^ host. Illumina sequencing of the leader-proximal spacer in CRISPR 1 allowed us to sample over 10^6^ acquired spacers in each replicate experiment (electronic supplementary material, figure S2 and table S6). In order to accurately map the spacers to the PLE 1 *V. cholerae* host, we performed complete whole-genome sequencing and assembly of the bacterial genome (electronic supplementary material, File S1). As was previously reported [12], we found that PLE 1 was integrated in a *V. cholerae* repeat, of which over 100 repeats intersperse the *V. cholerae* small chromosome in a gene-capture region, the superintegron [30]. In total, 96% of the acquired spacers mapped to PLE (electronic supplementary material, figure S3), while, interestingly, the other 4% mapped to *V. cholerae* chromosomes (electronic supplementary material, table S6).

**Figure 3.**
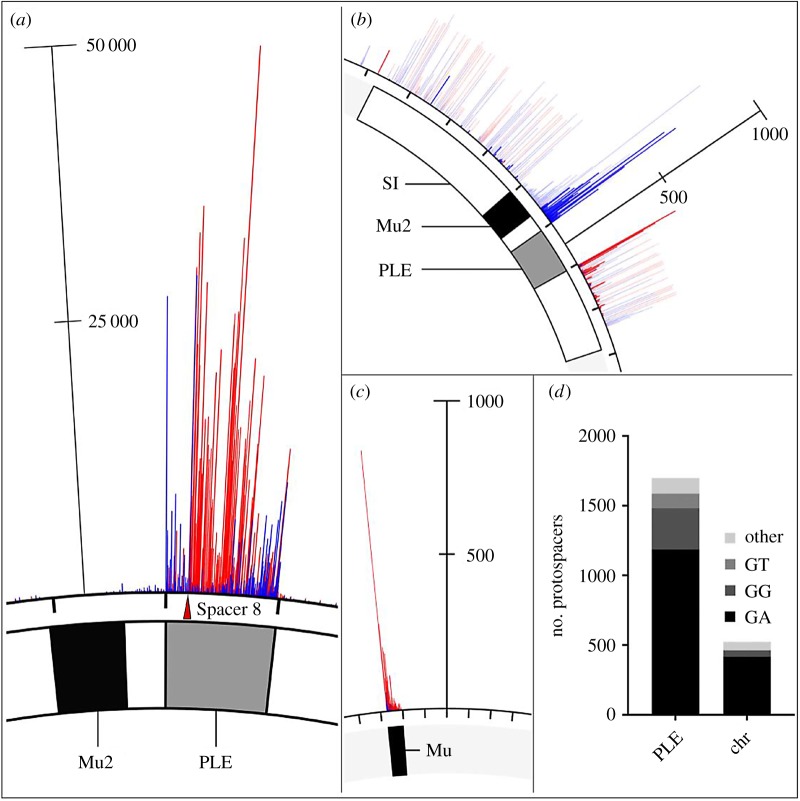
High-throughput interference-driven spacer acquisition mapping. (*a*) The locations of the ICP1 CRISPR leader-proximal spacer on the *V. cholerae* small chromosome. The location of the interference-efficient spacer (S8) is indicated with the red triangle. (*b*) Spacer locations on the *V. cholerae* small chromosome (PLE mappings not shown for clarity). Uniquely mapped spacers are shown in solid blue or red, while translucent bars show mapping of spacers to all possible locations. (*c*) Spacer locations on the *V. cholerae* large chromosome. For panels (*a–c*), spacers on the plus and minus strand are indicated in red and blue, respectively. The scale bar measures the number of mapped spacers, and the tick marks around the chromosome are in 18 kb intervals. The white box represents the superintegron (SI), the black box is the mu-like region and the grey box is PLE 1. (*d*) Proportion of unique protospacers with a GA or other dinucleotide PAM sequence in PLE or in the small chromosome (chr).

Mapping of the spacers to the small chromosome showed a pattern of strand bias that reflected previous observations in primed acquisition experiments performed in other Type I-F systems [31], with a distribution of acquired spacers 5′ of the protospacer on the non-targeted strand and 3′ of the protospacer on the targeted strand (figure 3*a*). The distribution of spacers acquired 5′ of the protospacer on the non-targeted strand were split between the small chromosomal region proximal to the PLE 1 integration site (figure 3*b*, electronic supplementary material, figure S4), as well as the 3′ end of PLE. Acquired spacers mapping to the *V. cholerae* chromosome were not evenly distributed between the large and small chromosome, but instead approximately 90% of the chromosomal spacers mapped to the small chromosome (figure 3*b*; electronic supplementary material table S6). Spacers that mapped to the large chromosome were restricted to a mu-like region (figure 3*c*), which was duplicated in this strain and was also in the small chromosome proximal to PLE (figure 3*b*). Acquired spacers mapped uniformly throughout the superintegron, however, this is likely an artefact as the superintegron is highly repetitive. When considering spacers that map to a single site in the small chromosome, we observed an obvious bias for acquired spacers mapping closer to the PLE integration site (figure 3*b*; electronic supplementary material, figure S4).

**Figure 4.**
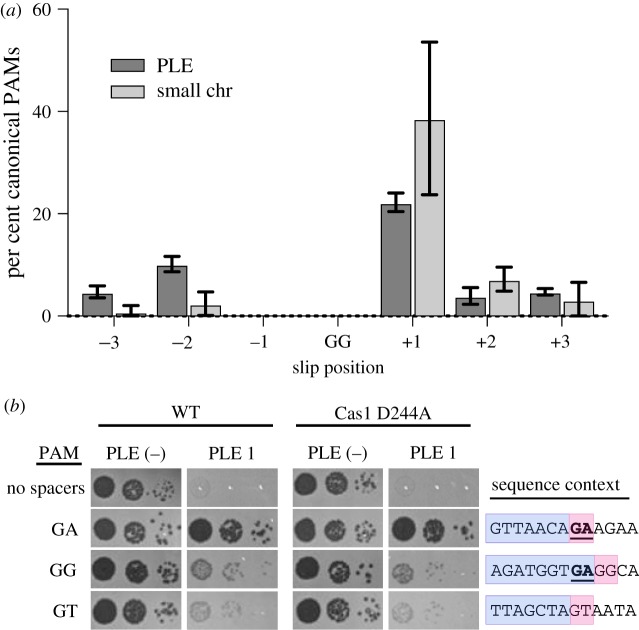
Characterizing non-canonical PAMs. (*a*) The frequency of a canonical GA PAM ± 3 nt from a non-canonical GG PAM across all data sets. (*b*) Tenfold dilutions of ICP1 engineered to contain a spacer that targets PLE 1 with a non-canonical PAM spotted on *V. cholerae* PLE (−) or PLE 1 lawns showing the ability of different phage strains to form plaques (dark spots, zones of killing) (left). Sequence context (right) of the region adjacent to the PAM. The protospacer is boxed in purple and PAM is boxed in pink. The consensus canonical PAM GA is bolded and underlined.

Consistent with CRISPR^+^ ICP1 isolates from nature (electronic supplementary material, figure S5), the majority (approx. 70%) of the spacers acquired experimentally targeted protospacers in PLE 1 that were flanked by a 3′ GA PAM (figure 3*d*). Variations from the canonical GA motif would be expected to abolish CRISPR interference. However, approximately 30% of protospacers in PLE had non-canonical PAMs, and of those, the majority were GG or GT. Previous CRISPR acquisition studies in Type I-F systems indicate that alternative PAMs can be explained by a ‘slippage’ event [31,32]. To identify putative slippage events, we analysed the sequences adjacent to GG PAMs and found that 45% of GG PAMs have a canonical GA within three nucleotides of the PAM position, suggesting that the ICP1 acquisition machinery has a propensity to slip (figure 4*a*).

We next wanted to determine if these non-canonical PAMs are functional for PLE interference. Since only the newest spacer was sequenced in our high-throughput assay, we could not rule out that multiple spacers were not acquired within the expanded phage CRISPR array. We therefore engineered ICP1 to encode a single spacer reflective of an experimentally acquired spacer with either the canonical PAM or the most common non-canonical PAMs: either a GG or GT (figure 3*d*), and evaluated plaque formation of the engineered phage on PLE 1 *V. cholerae*. Despite relying on a non-canonical PAM, we found that ICP1 is able to target those protospacers and overcome PLE, albeit at a lower efficiency than when targeting a protospacer with a canonical GA PAM (figure 4*b*). Even when no canonical PAM was within ± 3 nt, ICP1 was still able to overcome PLE targeting a protospacer with a GT PAM. As PAM mutations are frequently a source for primed acquisition [33], we tested if the observed residual CRISPR activity was due to further spacer acquisition and interference. We constructed a Cas1 D244A mutation, which disrupts a conserved metal coordinating residue to inhibit spacer acquisition [32] (electronic supplementary material, figure S6), and tested if plaque formation was altered (figure 4*b*). We observed no difference in the efficiency of plaque formation between the Cas1 mutants and the parental phage, suggesting that the ICP1 CRISPR-Cas system is more tolerant of divergent PAMs during infection than previously characterized [13].

### (d) Protospacers in the small chromosome facilitate ICP1 CRISPR-Cas-mediated PLE interference

**Figure 5.**
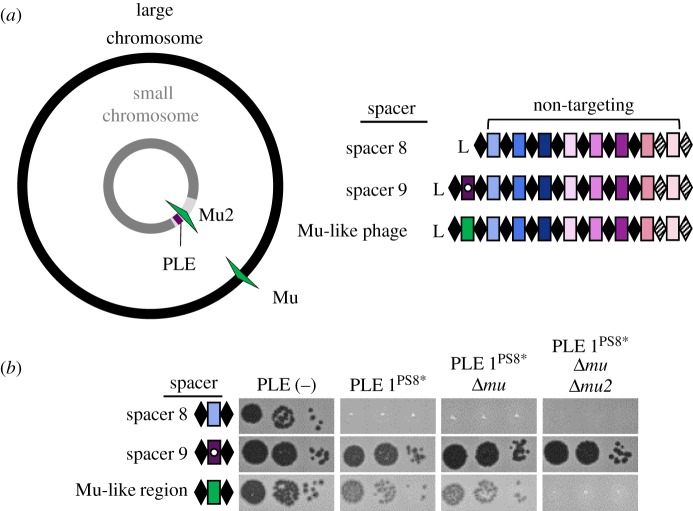
ICP1 CRISPR-targeting of the small chromosome facilitates PLE interference. (*a*) Cartoon (left) of the *V. cholerae* large and small chromosomes. The superintegron is shown in light grey, the PLE is shown in purple. The two mu-like regions in the large and small chromosome are shown by green arrows. ICP1_2011_A CRISPR variants (right) used to test the role of targeting sites. The internal white circle indicates the PLE 1 targeting spacer. (*b*) Tenfold dilutions of ICP1 with the spacers indicated spotted on *V. cholerae* lawns showing the ability of different phage strains to form plaques.

In our spacer acquisition experiment, we identified a subset of spacers that target a mu-like region in the *V. cholerae* large chromosome (figure 3*c*), suggesting that CRISPR targeting of the mu-like region was advantageous in overcoming PLE. To test the role of protospacers in the mu-like region in PLE interference, we isolated ICP1 that had acquired a spacer that targets the mu-like region and was able to form plaques on PLE 1^PS8*^ (figure 5). Since assembly of the *V. cholerae* genome revealed that the mu-like region was present and 100% identical in both chromosomes, presumably due to a duplication of the region on the large chromosome (figure 5*a*), we wanted to evaluate if targeting the mu-like region *per se* allowed for plaque formation, or if the chromosomal context was important in allowing for CRISPR-meditated interference with PLE. To test this difference, we generated a single knockout of the mu-like region in the large chromosome and a double knockout in both chromosomes. ICP1 CRISPR-mediated interference with PLE was abolished in the double knockout, however, knocking out the mu-like region in the large chromosome had no effect on ICP1 plaque formation, demonstrating that targeting of the region in the large chromosome is an artefact of the duplication in the small chromosome (figure 5*b*). These results show that CRISPR targeting of the *V. cholerae* large chromosome is dispensable for phage overcoming PLE, while targeting the small chromosome is sufficient to overcome PLE activity.

### (e) When CRISPR goes off target: going the distance to maintain interference

**Figure 6.**
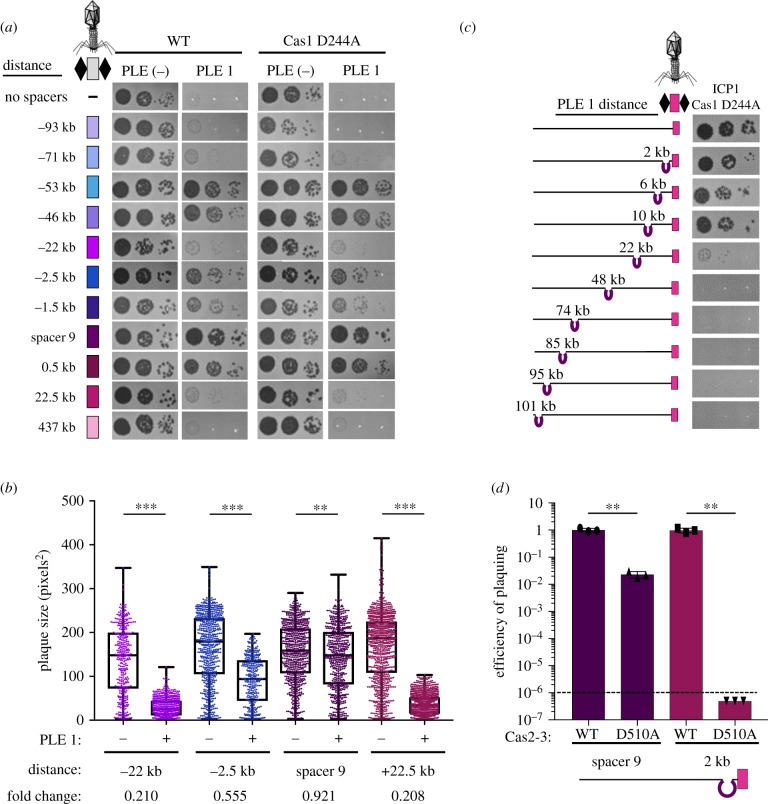
The interference potential of spacers directed to the small chromosome is dependent on the proximity to PLE. (*a*) Tenfold dilutions of ICP1 engineered with a spacer that targets the small chromosome or PLE 1 +/− Cas1 spotted on lawns of *V. cholerae* showing the ability of different phage strains to form plaques. Spacer 9 is the same as spacer 9 in ICP1_2011_A. The distance of the chromosomal protospacer from the PLE 1 integration site is indicated*.* (*b*) Plaque size of ICP1 variants plated with *V. cholerae*. The distance and colour scheme correspond to the spacers tested in (*a*). The fold change in average size of a plaque on a PLE (+) host compared to a PLE (−) host is indicated at the bottom. Significance was determined by Mann–Whitney *U*-test, **p < 0.005, ***p < 0.0001. (*c*) Tenfold dilutions of ICP1 engineered with a chromosomal protospacer spotted on lawns of *V. cholerae* harbouring PLE in different locations in the small chromosome. (*d*) Efficiency of plaquing of phage engineered to contain a spacer that is internal to PLE 1 (spacer 9) or targeting the small chromosome 2 kb away from PLE 1 (same as in (*c*), cartoon below graph) with the WT or helicase dead (D510A) Cas2-3 allele. The dashed line indicates the limit of detection. Significance was determined by *T*-test, **p < 0.005.

As processivity of Cas2-3 has been demonstrated *in vitro* [17], we speculated that ICP1 targeting of the small chromosome proximal to PLE interferes with PLE anti-phage activity by the processive degradation of PLE along with the chromosome; however, PLE excises from the chromosome early during ICP1 infection [20]. This timing suggests that CRISPR targeting and Cas2-3 processive degradation of the small chromosome would have to happen prior to PLE excision and would therefore likely be distance dependent. In support of this hypothesis, experimentally acquired spacers mapping to the small chromosome clustered proximal to PLE (figure 3*b*). To test the impact of targeting at increasing distances from PLE, we engineered ICP1 to possess CRISPR arrays containing only one spacer drawn from the experimental acquisition pool that targets the small chromosome at varying distances away from PLE. We then assayed the ability of this engineered phage to overcome PLE and form plaques (figure 6*a*). As a positive control, ICP1 engineered with a spacer that targets internal to PLE formed robust and equal plaques on PLE(-) and PLE 1 hosts. In comparison, phage with a spacer that targets far (greater than 400 kb) from PLE were unable to form plaques on PLE 1. Conversely, ICP1 that target a protospacer only 0.5, 1.5 or 2.5 kb from PLE were able to efficiently overcome PLE and form plaques. Phage targeting protospacers at intermediate distances away from PLE (greater than 20 kb) demonstrated weak plaque formation on PLE 1. Surprisingly, we observed that ICP1 with some spacers targeting relatively far from PLE (53 and 46 kb away) were still able to form robust plaques on PLE 1 (figure 6*a*). While all of the spacers selected for this assay had one perfect protospacer match in the chromosome and have a GA PAM, we identified greater than 100 promiscuous putative target sites for these spacers which would bring the chromosomal target much closer to PLE 1 (electronic supplementary material, table S7), which may explain these phage's ability to overcome PLE. To test if spacer acquisition had a role in plaque formation, we engineered the chromosomal targeting phage in a Cas1-deficient background and assayed for plaque formation on the PLE 1 host. Despite being unable to acquire spacers (electronic supplementary material, figure S6), the Cas 1 = deficient phage retained the same plaquing phenotype. We quantified the weaker plaque formation observed when ICP1 targets 2.5 kb and greater than 20 kb away from PLE 1 by measuring plaque size compared to PLE (−) *V. cholerae* (figure 6*b*). As compared to phage with PLE internal and PLE proximal spacers, phage with chromosomal spacers targeting greater than 20 kb away from PLE had significantly limited plaque size; however, even phage with a chromosomal spacer that is proximal to PLE has an approximately 50% smaller plaque size when compared to plaques on a PLE (−) host. These results indicate that some PLE-mediated anti-phage activity is retained when CRISPR-Cas is directed at increasing distances from PLE in the small chromosome, but direct targeting of PLE is still required for maximizing phage fitness.

To control for differences in spacer sequences, we also varied the location of the PLE and tested the ability of ICP1 with a single spacer targeting the small chromosome to interfere with PLE 1. Following ICP1-mediated transduction, PLE 1 integrates into a *V. cholerae* repeat in the new host [12]. We collected a pool of PLE 1 transductants where PLE was integrated at varying distances from the chromosomal protospacer and challenged these strains with ICP1. As a control, we determined that all of the tested PLE 1 *V. cholerae* hosts were susceptible to ICP1 CRISPR-Cas interference when ICP1 possessed a PLE internal spacer (electronic supplementary material, figure S7a). Consistent with our earlier finding, PLE integrated at an increasing distance away from the protospacer was less susceptible to ICP1-encoded CRISPR interference (figure 6*c*).

**Figure 7.**
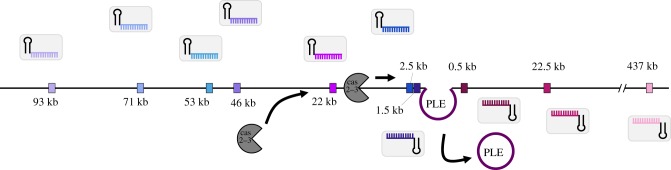
Model of race between ICP1 Cas2-3 processive degradation of the *V. cholerae* chromosome and ICP1-mediated PLE excision. Csy complexes (grey boxes) with crRNAs (coloured) search for a complementary protospacer (coloured rectangles, experimentally assessed in figure 6*a*). Cas2-3 (dark grey) is recruited to the protospacer and processively degrades the DNA towards PLE (purple). ICP1 is able to form plaques when Cas2-3 degrades PLE before PLE excises from the chromosome, which occurs within 5 min of ICP1 infection.

As Cas2-3 has been demonstrated to translocate *in vitro,* we next wanted to see if the indirect inhibition of PLE by spacers that target the small chromosome was due to Cas2-3 processivity. We constructed a Cas2-3 helicase dead variant by mutating the conserved DExx helicase motif II (D510A) [16] (electronic supplementary material, figure S7b) and tested the ability of ICP1 to form plaques on PLE 1 *V. cholerae*. ICP1 Cas2-3 D510A engineered with a spacer that targets internal to PLE was still able to form plaques on a PLE (+) host, although both the efficiency of plaquing and plaque size were negatively impacted by the helicase mutation (figure 6*d*; electronic supplementary material, figure S7c). Conversely, when ICP1 targets the *V. cholerae* small chromosome 2 kb away from PLE, the helicase activity of ICP1 Cas2-3 was absolutely essential for PLE interference and plaque formation (figure 6*d*; electronic supplementary material, figure S7c). These findings are the first direct demonstration of functional Cas2-3 processivity *in vivo* and support our model of indirect targeting (figure 7).

## 4. Discussion

Our results reveal that the latest front in the ongoing arms race between contemporary isolates of epidemic *V. cholerae* and its predator ICP1 necessitate the persistence of the ICP1-encoded Type I-F CRISPR-Cas system to counter PLE-mediated anti-phage activity (figure 1). By using a high-throughput spacer acquisition assay, we gained insight into the full range of spacers that can combat PLE. Interestingly, our experimental findings on acquisition and interference do not reflect the rather limited diversity of spacers that ICP1 maintains against PLE in nature. These results highlight that not all spacers are equally proficient for interference, and that coupled analysis of these competing mobile genetic elements from nature reveals the evolutionary benefits of a particular complement of spacers more so than laboratory-based studies. Despite a lack of clear evidence indicating where the ICP1-encoded CRISPR-Cas system originated, it serves as a tractable model through which we can examine the biology of an endogenous Type I-F CRISPR-Cas system against its cognate foe.

Co-culture studies competing phage against CRISPR-Cas proficient bacterial hosts demonstrated that mutational escape by phage is limited by bacterial populations that have heterogenous CRISPR arrays [34]. Here, we see that PLE 1 is highly conserved over time, even when co-circulating with CRISPR proficient ICP1. In the light of previous suggestions, the diversity of CRISPR arrays in ICP1 populations may limit the success of PLE escape mutants. Surprisingly, however, we see very little diversity in the spacer composition of ICP1 CRISPR arrays with the same minimal spacers being conserved in phage circulating for over 8 years (figure 1*b*). Likewise, CRISPR-proficient ICP1 isolated from nature always encoded more than one spacer against PLE, which would be expected to limit CRISPR escape mutations. It may be that there is limited room for genetic drift in the PLE genome, permitting ICP1 to streamline its CRISPR array, keeping only the most efficient spacers while also maintaining an advantageous genome size.

Akin to studies of bacterial Type I-F CRISPR-Cas mediated interference with plasmid transformation and conjugation [35], we similarly see that the spacer sequence and quantity of spacers in the array have a role in ICP1's ability to abolish PLE spread (figure 2). This may be due to differences in crRNA abundance or stability, or sequence-dependent subtleties that dictate interference potential, as has been proposed previously [36]. Despite spacer 9's improved interference with PLE mobilization compared to spacer 8, we still observed a slight defect in plaque size when comparing engineered phage with only spacer 9 relative to a PLE (−) host (figure 6*b*), suggesting that even this improved spacer alone is not sufficient to fully overcome PLE-mediated anti-phage activity. By encoding a seemingly redundant set of spacers targeting PLE, ICP1 increases its ability to overcome PLE and limit PLE spread in the environment. Additionally, multi-site targeting of *V. cholerae* PLE by ICP1 CRISPR-Cas may contribute to the modular evolution observed between PLE variants and dictate which PLEs are circulating within and between epidemics [12].

As expected, the majority of spacers acquired in our high-throughput acquisition assay directly target PLE (figure 3*a*). Analysis of natural ICP1 isolates recovered from cholera patient stool samples shows that the phage-encoded CRISPR-Cas system recognizes a GA PAM (electronic supplementary material, figure S4) which, although atypical for Type I-F systems [37], has been confirmed through single mutations to a C in both positions [13]. Notably, we found that ICP1 was able to incorporate spacers that targeted non-canonical PAMs (figure 3*d*) and that these spacers can suffice for PLE interference (figure 4*b*). In comparison to another high-throughput spacer acquisition assay in a Type I-F system, which found greater than 90% of all protospacers flanked by the canonical PAM [31], it appears that the phage-encoded system is less discriminating with only 70% of protospacers flanked by the expected PAM. However, targeting a protospacer with a non-canonical PAM reduced the ability of ICP1 to form plaques compared to the canonical PAM (figure 4*b*). As such, in nature ICP1 targeting a protospacer with a non-canonical PAM would not be able to completely interfere with PLE and thus would be selected against. This hypothesis is additionally supported by the observation that very few non-canonical PAM protospacers were associated with indirect targets in the small chromosome. As these chromosomal spacers are themselves less proficient for interference (figure 6*a,b*), the added disadvantage of targeting a protospacer with a non-canonical PAM likely tips the balance in favour of PLE, possibly explaining the lower abundance of these spacers in our selection experiments.

Despite the presence of spacers that target the *V. cholerae* large chromosome in the high-throughput spacer acquisition assay (figure 3*c*), we show that targeting this chromosome is dispensable for CRISPR interference of PLE and is likely an artefact of a duplication event of the mu-like region in the strain used in our assays (figure 5). Interestingly, two of the natural ICP1 isolates contain a spacer that targets a gene on the *V. cholerae* large chromosome (figure 1*b*). We speculate that this spacer was acquired from a *V. cholerae* strain possessing a duplication or rearrangement that is not represented in currently sequenced isolates, in which the protospacer was in the small chromosome proximal to PLE, allowing the phage to overcome PLE activity. However, this spacer does not seem to be maintained in the phage population, likely due to diminished PLE interference relative to PLE-direct spacers as we experimentally observed.

CRISPR targeting of the *V. cholerae* small chromosome can overcome PLE, but our results suggest a model in which there is a limit to the distance over which processive Cas2-3 degradation can occur to reach the PLE prior to excision (figure 7), an action which occurs within 5 min of ICP1 infection that is directed by an early expressed ICP1 protein [20]. The limit of processivity appears to be around a distance of 23 kb (figure 6*a,c*), at which point either Cas2-3 is unable to continue to process along the *V. cholerae* chromosome or PLE excises before interference occurs. *In vitro* studies of Cas3 from Type I-E systems have demonstrated Cas3 translocation velocities of 89–300 bases per second and average processivities between 12 and 19 kb [38,39], however, the functional role and limitations of processivity *in vivo* are not known. Our results are the first to demonstrate that Cas2-3 is processive *in vivo,* with over 22 kb from a distal chromosomal protospacer over which the ICP1 CRISPR-Cas can maintain activity to overcome PLE when Cas2-3 has the ability to translocate along DNA (figure 6*d*). As this event must occur within 5 min of ICP1 initiating infection, the estimated processivity of ICP1 Cas2-3 is within the range of what has been reported for Type I-E Cas3, which is especially remarkable given the complexity of the crowded intracellular environment compared to simplified *in vitro* systems.

In comparison to other Cas nucleases like Cas9, which introduces a single double-stranded break [40,41], Cas2-3 degrades DNA as it translocates away from the protospacer [17], making it more likely to destroy and thus interfere with its target. In fact, we see that the helicase dead Cas2-3 is less able to overcome PLE even when directly targeting the anti-phage island, suggesting that the processive degradation of PLE contributes to interference (figure 6*d*). Similarly, this predicted advantage may account for the increased prevalence of Type I systems for phage defence [42]. In the context of the battle between ICP1 and PLE, this processivity permits interference even with an indirect CRISPR target and has important implications for harnessing CRISPR-Cas in biotechnology and medicine. Since the characterization of the ICP1-encoded CRISPR-Cas system, phage engineered with CRISPR-Cas systems to target virulent, antibiotic-resistant bacteria have been assayed for therapeutic applications [43,44], showing the value of innovating from natural systems to overcome disparate biological problems.

## Supplementary Material

Supplementary material

## Supplementary Material

KDS1 genome

## Data accessibility

The datasets supporting this article have been uploaded as part of the electronic supplementary material. The sequencing data have been deposited in the Sequence Read Archive database under accession codes SRR7826356, SRR7826357, SRR7826358, SRR7827053, SRR7827054 and SRR7827055.
