# Supplementary material for: Competition between mobile genetic elements drives optimization of a phage-encoded CRISPR-Cas system: insights from a natural arms race
Source: Philos Trans R Soc Lond B Biol Sci. 2019 Mar 25;374(1772):20180089. doi: 10.1098/rstb.2018.0089 (PMC6452262; doi:10.1098/rstb.2018.0089)
Supplement: Supplementary material [file rstb20180089supp1.docx]

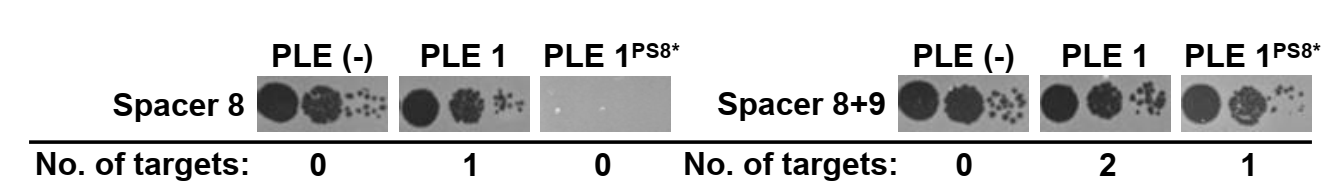


**Supplementary Figure 1. A single spacer is necessary and sufficient to permit lytic growth of ICP1 on PLE 1 *V. cholerae* as seen by equal plaque formation.** Tenfold dilutions of ICP1 with the indicated spacers were spotted on the labeled *V. cholerae* lawns. PS8* indicates silent mutations in protospacer 8 that abolishes CRISPR interference (1). The number of functional protospacers are listed.


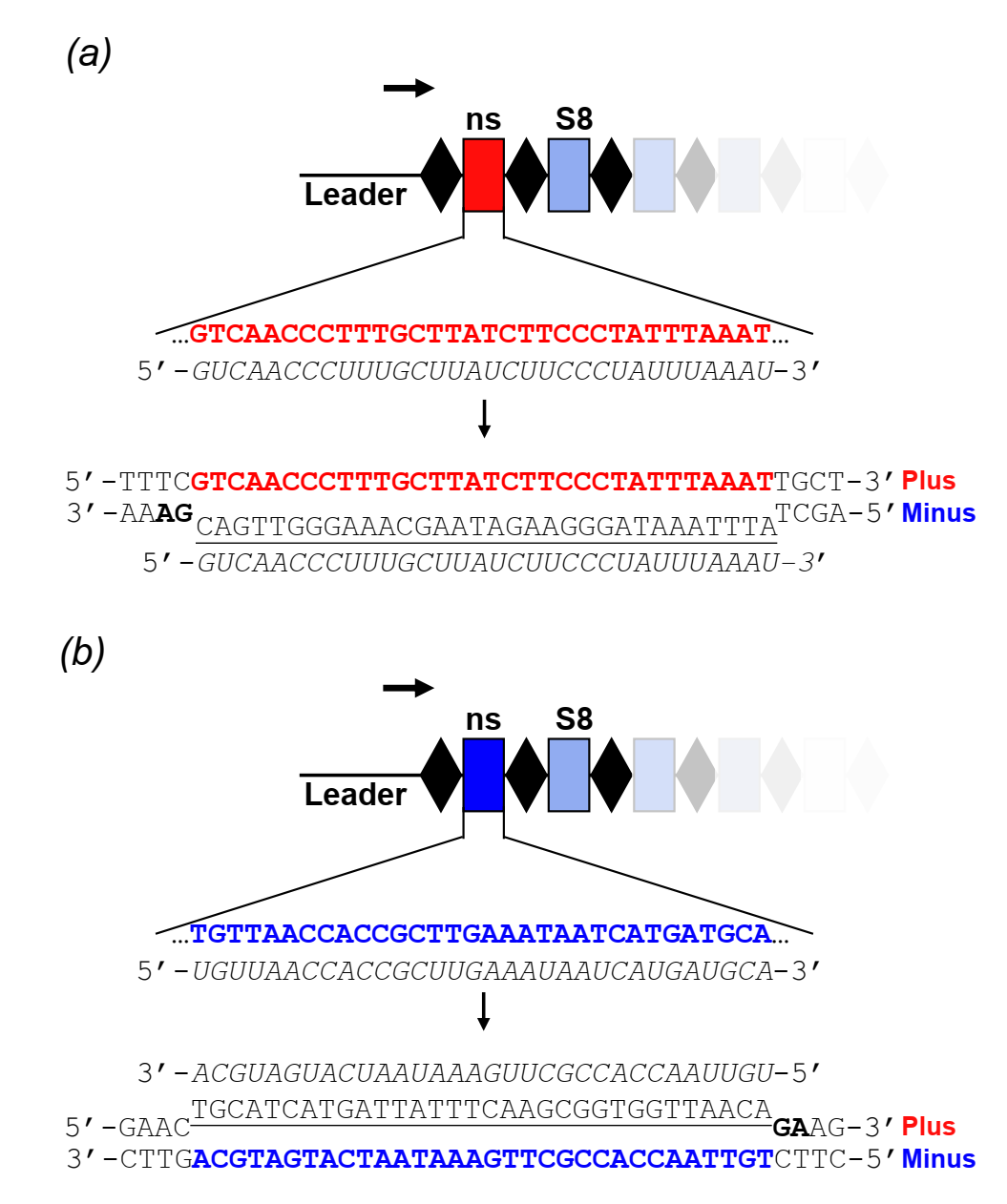


**Supplementary Figure 2. Schematic of high-throughput spacer acquisition mapping to the *V. cholerae* chromosome(s).** The newest spacer from the ICP1 CRISPR array was sequenced using the oligo indicated in the black horizontal arrow, with the 50bp single end illumine read initiating from that arrow. The new spacer either matches to the plus strand *(a)* or the minus strand *(b)*. The spacer DNA sequence is bolded and colored, the crRNA is italicized, the protospacer is underlined and the PAM is bolded in black.
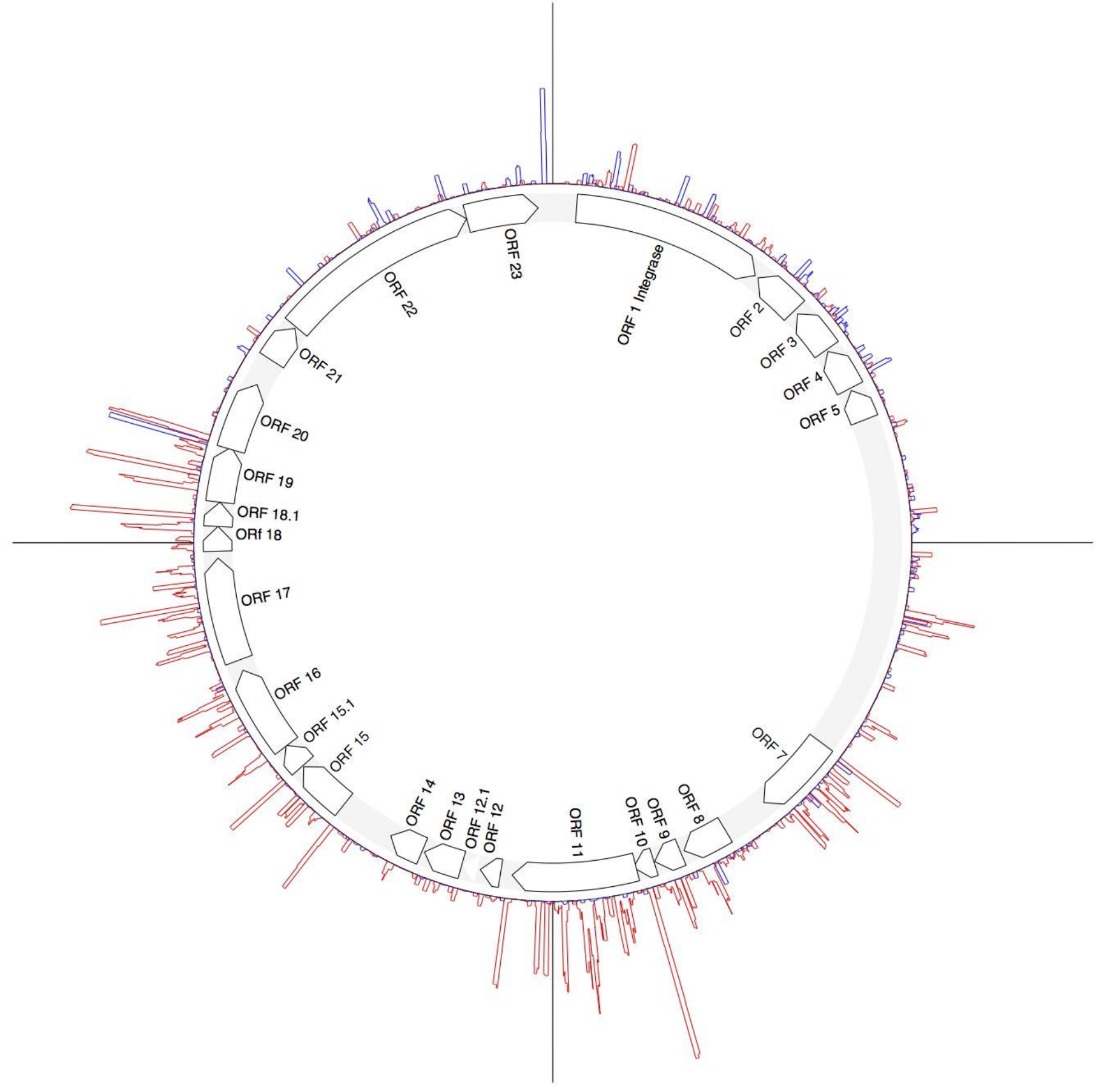


**Supplementary Figure 3. High-throughput spacer acquisition mapping on circularized PLE 1.** Spacers which uniquely mapped to PLE 1 on the plus or minus strand are indicated in red and blue, respectively. Black scale-bars at each quadrant represent a height of 50,000 spacer hits. The PLE 1 integration location is at the 12 o’clock scale-bar position. Putative open reading frames are indicated by white arrows. This representative graph displays results from replicate 2 in Supplementary Fig. 4.


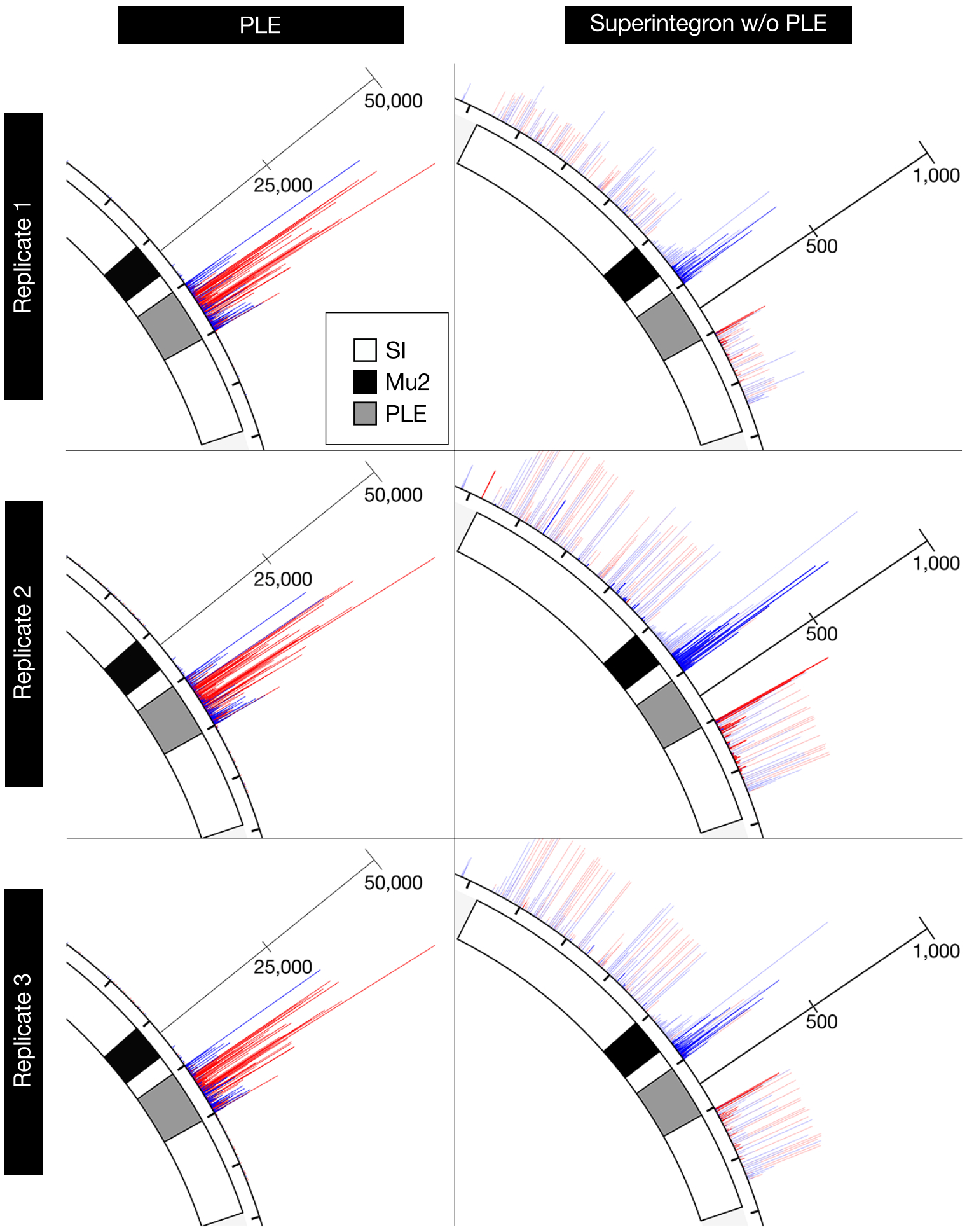


**Supplementary Figure 4. Replicates of high-throughput ICP1 spacer acquisition mapping to the small chromosome**. Spacer locations of the most leader-proximal spacers on the plus and minus strand are indicated in red and blue, respectively. Uniquely mapped spacers are shown in solid blue or red, while translucent bars show mapping of spacers to all possible locations. The scale bars measure the number of mapped spacers, and the tick marks around the chromosome are in 18kb intervals. Replicate 2 is the same data as in Fig. 3a and b and Supplementary Fig. 3.


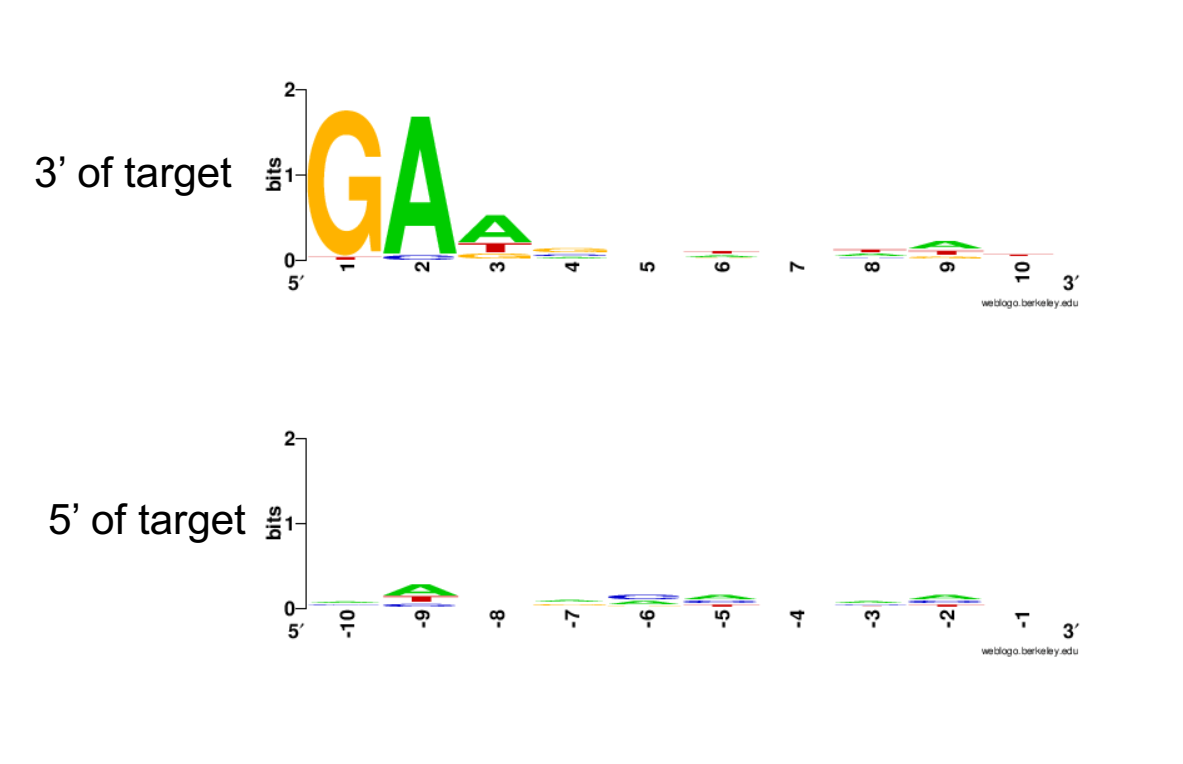


**Supplementary Figure 5. Sequence logo of PAMs of natural ICP1 isolates.** The PAM sequence of the ICP1-encoded CRISPR-Cas system. Alignment of flanking sequence of all known targets of spacers found in natural ICP1 isolates. Sequence logos were generated using WebLogo (2).


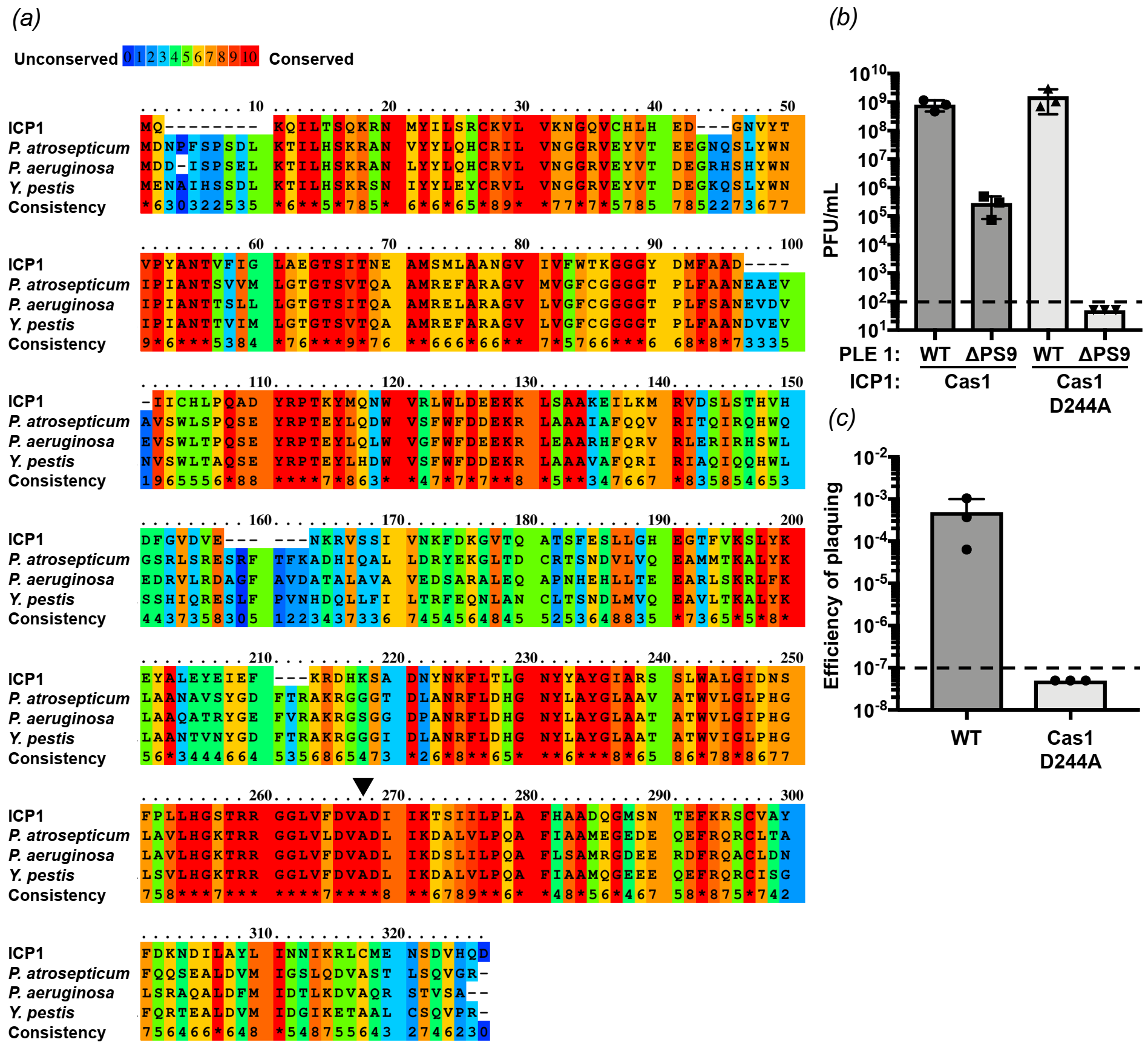


**Supplementary Figure 6.** **Conserved residue in ICP1’s Cas1 is necessary for spacer acquisition.** *(a)* Praline alignment (3) of Cas1 from ICP1 and other Type I-F systems in the organism indicated. The black arrow indicates the conserved residue mutated in ICP1 (D244A). *(b,c)* Engineered phage with a PLE internal spacer (S9) +/- Cas1 D244A were passaged on a PLE(+) host. Ten plaques of each phage were picked to generate a phage stock, and each stock was re-plaqued onto a PLE(+) host and a PLE 1^ΔPS9^ host (as plaque formation on the PLE 1^ΔPS9^ host requires spacer acquisition) and the number of plaques were counted *(b)*. The efficiency of plaquing *(c)* is calculated as the number of plaques on a PLE ^ΔPS9^ host relative to the number of plaques on a WT PLE(+) host. Plaque formation on the PLE 1^ΔPS9^ could not be detected in the Cas 1 D244A mutant (the dashed line indicates the limit of detection), compared to the WT phage in which approximately 1 phage per 2,835 acquired a new spacer that enabled plaque formation on the PLE 1^ΔPS9^ host (EOP =3.3x10^-4^). Error bars indicate standard deviation of three independent replicates.


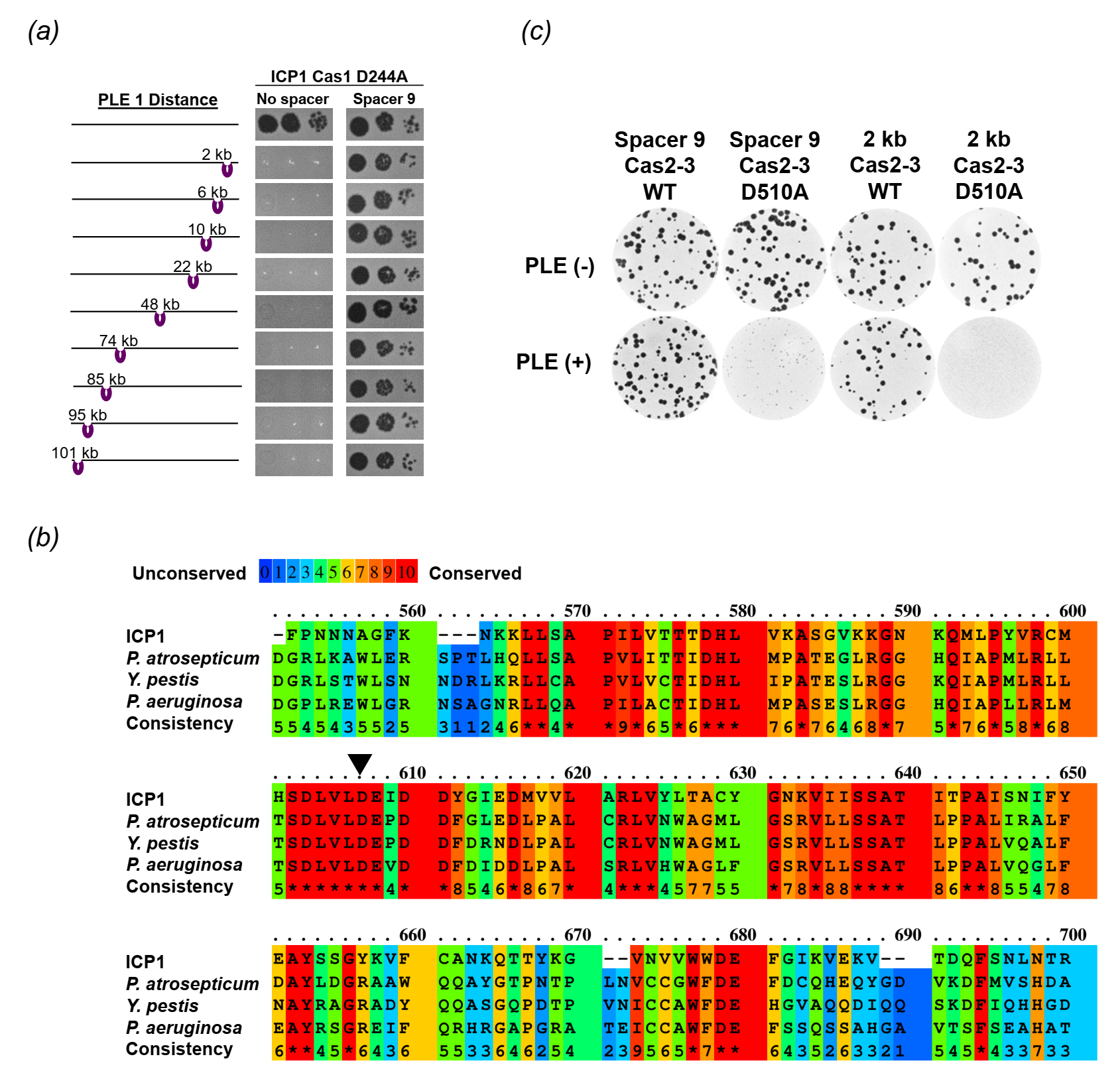


**Supplementary Figure 7. PLE transduced in unique sites in *V. cholerae* chromosome can be overcome by ICP1-CRISPR mediated interference dependent upon Cas2-3 helicase activity.** *(a)* Tenfold dilutions of ICP1 without any spacer or with a PLE targeting spacer (S9) spotted on lawns of *V. cholerae*. Transductants used are the same as in Figure 6c. *(b)* Praline alignment (3) of a region of Cas2-3 from ICP1 and other Type I-F systems in the organism indicated. The black arrow indicates the conserved residue mutated in ICP1 (D510A). *(c)* Plaque plates of different concentrations of ICP1 with the labeled spacer in the WT or helicase dead (D510A) Cas2-3 background. The concentration of the 2 kb Cas2-3 D510A phage is so high that *V. cholerae* is unable to form a healthy lawn, but no plaques were detected.

**Supplementary Table S1: Phage used in this study**

| Phage | Description | Source |
| --- | --- | --- |
| ICP1_2011_A | CRISPR-Cas positive ICP1 isolate recovered from cholera patient stool with KS393 | (1) |
| ICP1_2001_E | CRISPR-Cas positive ICP1 isolated collected from water sample in Bangladesh, referred to as JSF5 in original publication | (4) |
| ICP1_2001_F | CRISPR-Cas positive ICP1 isolated collected from water sample in Bangladesh, referred to as JSF6 in original publication | (4) |
| ICP1_2005_A | CRISPR-Cas positive ICP1 isolate from cholerae patient stool collected at ICDDR,B | (5) |
| ICP1_2006_E | CRISPR-Cas positive ICP1 isolate from cholerae patient stool collected at ICDDR,B | (6) |
| ICP1_2004_A | CRISPR-Cas positive ICP1 isolate from cholerae patient stool collected at ICDDR,B | (5) |
| ICP1_2009_A | CRISPR-Cas positive ICP1 isolated collected from water sample in Bangladesh, referred to as JSF13 in original publication | (4) |
| ICP1_2011_B | CRISPR-Cas poitive ICP1 genome assembled from metagenomic analysis of diarrheal stool sample from ICDDR,B | (6) |
| ICP1_2011_C | CRISPR-Cas positive ICP1 isolated collected from water sample in Bangladesh, referred to as JSF14 in original publication | (4) |
| ICP1_2012_B | CRISPR-Cas positive ICP1 isolated collected from water sample in Bangladesh, referred to as JSF17 in original publication | (4) |
| ICP1_2015_A | CRISPR-Cas positive ICP1 isolate recovered from cholera patient stool collected from ICDDR,B | This study |
| ICP1_2016_A | CRISPR-Cas positive ICP1 isolate recovered from cholera patient stool collected from ICDDR,B | This study |
| ICP1_2017_A | CRISPR-Cas positive ICP1 isolate recovered from cholera patient stool collected from ICDDR,B | This study |
| ICP1_2017_B | CRISPR-Cas positive ICP1 isolate recovered from cholera patient stool collected from ICDDR,B | This study |
| ICP1_2017_C | CRISPR-Cas positive ICP1 isolate recovered from cholera patient stool collected from ICDDR,B | This study |
| ICP1_2017_D | CRISPR-Cas positive ICP1 isolate recovered from cholera patient stool collected from ICDDR,B | This study |
| ICP1_2017_E | CRISPR-Cas positive ICP1 isolate recovered from cholera patient stool collected from ICDDR,B | This study |
| ICP1_2017_F | CRISPR-Cas positive ICP1 isolate recovered from cholera patient stool collected from ICDDR,B | This study |
| ICP1_2011_A ΔS9 | Spontaneous deletion of spacer 9 in phage CRISPR array | (1) |
| ICP1_2011_A S10 | ICP1_2011_A with expanded CRISPR array with new spacer against PLE in leader proximal CRISPR 1 array, isolated from selection of ICP1_2011_A on PLE PS8* PS9* host | This study |
| ICP1_2011_A ΔS2-9 | Directed deletion of spacers 2-9 in phage CRISPR array | (7) |
| ICP1_2011_A ΔS9, new mu spacer | ICP1_2011_A ΔS9 with new spacer against mu-like region in leader proximal CRISPR 1 array, isolated from high-throughput spacer acquisition pool | This study |
| ICP1_2011_A ΔS2-9, Cas1D244A | Engineered Cas1D244 mutation to prevent spacer acquisition in ICP1_2011_A | This study |
| ICP1_2011_A -93kb (+/- Cas1D244A) | ICP1_2011_A ΔS2-9 engineered with pCRISPR editing template for custom spacer selected from acquisition pool at a specific distance from PLE 1. Constructed in ΔS2-9 and ΔS2-9 Cas1D244A background | This study |
| ICP1_2011_A -71kb (+/- Cas1D244A) | ICP1_2011_A ΔS2-9 engineered with pCRISPR editing template for custom spacer selected from acquisition pool at a specific distance from PLE 1. Constructed in ΔS2-9 and ΔS2-9 Cas1D244A background | This study |
| ICP1_2011_A -53kb (+/- Cas1D244A) | ICP1_2011_A ΔS2-9 engineered with pCRISPR editing template for custom spacer selected from acquisition pool at a specific distance from PLE 1. Constructed in ΔS2-9 and ΔS2-9 Cas1D244A background | This study |
| ICP1_2011_A -46kb (+/- Cas1D244A) | ICP1_2011_A ΔS2-9 engineered with pCRISPR editing template for custom spacer selected from acquisition pool at a specific distance from PLE 1. Constructed in ΔS2-9 and ΔS2-9 Cas1D244A background | This study |
| ICP1_2011_A -22kb (+/- Cas1D244A) | ICP1_2011_A ΔS2-9 engineered with pCRISPR editing template for custom spacer selected from acquisition pool at a specific distance from PLE 1. Constructed in ΔS2-9 and ΔS2-9 Cas1D244A background | This study |
| ICP1_2011_A -2.5kb (+/- Cas1D244A) | ICP1_2011_A ΔS2-9 engineered with pCRISPR editing template for custom spacer selected from acquisition pool at a specific distance from PLE 1. Constructed in ΔS2-9 and ΔS2-9 Cas1D244A background | This study |
| ICP1_2011_A -1.5kb (+/- Cas1D244A) | ICP1_2011_A ΔS2-9 engineered with pCRISPR editing template for custom spacer selected from acquisition pool at a specific distance from PLE 1. Constructed in ΔS2-9 and ΔS2-9 Cas1D244A background | This study |
| ICP1_2011_A PLE Internal (S9) (+/- Cas1D244A) | ICP1_2011_A ΔS2-9 engineered with pCRISPR editing template for custom spacer selected from acquisition pool at a specific distance from PLE 1. Constructed in ΔS2-9 and ΔS2-9 Cas1D244A background | This study |
| ICP1_2011_A 0.5kb (+/- Cas1D244A) | ICP1_2011_A ΔS2-9 engineered with pCRISPR editing template for custom spacer selected from acquisition pool at a specific distance from PLE 1. Constructed in ΔS2-9 and ΔS2-9 Cas1D244A background | This study |
| ICP1_2011_A 22.5kb (+/- Cas1D244A) | ICP1_2011_A ΔS2-9 engineered with pCRISPR editing template for custom spacer selected from acquisition pool at a specific distance from PLE 1. Constructed in ΔS2-9 and ΔS2-9 Cas1D244A background | This study |
| ICP1_2011_A 437kb (+/- Cas1D244A) | ICP1_2011_A ΔS2-9 engineered with pCRISPR editing template for custom spacer selected from acquisition pool at a specific distance from PLE 1. Constructed in ΔS2-9 and ΔS2-9 Cas1D244A background | This study |
| ICP1_2011_A GA PAM (+/- Cas1D244A) | ICP1_2011_A ΔS2-9 engineered with pCRISPR editing template for custom spacer selected from acquisition pool at a specific distance from PLE 1. Constructed in ΔS2-9 and ΔS2-9 Cas1D244A background | This study |
| ICP1_2011_A GG (+/- Cas1D244A) | ICP1_2011_A ΔS2-9 engineered with pCRISPR editing template for custom spacer selected from acquisition pool at a specific distance from PLE 1. Constructed in ΔS2-9 and ΔS2-9 Cas1D244A background | This study |
| ICP1_2011_A GT (+/- Cas1D244A) | ICP1_2011_A ΔS2-9 engineered with pCRISPR editing template for custom spacer selected from acquisition pool at a specific distance from PLE 1. Constructed in ΔS2-9 and ΔS2-9 Cas1D244A background | This study |

**Supplementary Table S2: Bacterial strains used in this study**

| Strain | Description | Source |
| --- | --- | --- |
| KS393 | *V. cholerae* clinical Isolate from the ICDDR, B, harbors PLE 1 | (1) |
| KDS2 | KS393 PLE 1 PS8* such that target of ICP1_2011 CRISPR spacer 8 target is mutated | (1) |
| KDS3 | KS393 PLE 1 PS8*, Kanamycin resistance cassette inserted downstream of ORF 23 | (8) |
| KDS4 | KS393 PLE 1 Kanamycin resistance cassette inserted downstream of ORF 23 | (8) |
| KS1265 | KS393 ΔPLE 1:Spec. Natural transformation used to knockout PLE 1 and replace it with a spectinomycin cassette | This study |
| KDS46 | KDS3 PLE 1 Δorf3. Natural transformation used to knockout PLE 1 orf3 and replace it with a frt scar | This study |
| ACM71 | KDS2 Δmu. Natural transformation used to knockout mu-like region in large chromosome and replace it with a kanamycin cassette | This study |
| ACM146 | KDS2 Δmu, Δmu(2). Natural transformation used to knockout mu-like region in small chromosome in KS393 Δmu background and replace it with a spectinomycin cassette | This study |
| ACM584 | *V. cholerae* clinical Isolate from the ICDDR, B, harbors PLE 1 | This study |
| ACM585 | *V. cholerae* clinical Isolate from the ICDDR, B, harbors PLE 1 | This study |
| ACM589 | *V. cholerae* clinical Isolate from the ICDDR, B, harbors PLE 1 | This study |
| ACM592 | *V. cholerae* clinical Isolate from the ICDDR, B, harbors PLE 1 | This study |
| ACM596 | *V. cholerae* clinical Isolate from the ICDDR, B, harbors PLE 1 | This study |
| ACM598 | *V. cholerae* clinical Isolate from the ICDDR, B, harbors PLE 1 | This study |
| ACM604 | *V. cholerae* clinical Isolate from the ICDDR, B, harbors PLE 1 | This study |
| ACM606 | *V. cholerae* clinical Isolate from the ICDDR, B, harbors PLE 1 | This study |
| ACM608 | *V. cholerae* clinical Isolate from the ICDDR, B, harbors PLE 1 | This study |
| ACM612 | *V. cholerae* clinical Isolate from the ICDDR, B, harbors PLE 1 | This study |
| ACM614 | *V. cholerae* clinical Isolate from the ICDDR, B, harbors PLE 1 | This study |
| ACM618 | *V. cholerae* clinical Isolate from the ICDDR, B, harbors PLE 1 | This study |
| KS1559 | *V. cholerae* clinical isolate (PLE -) P*tac*-CRISPR-Cas from O395, KanR ΔlacZ:: SpecR, constructed as per Box *et al*, 2016 for engineering ICP1 phage using the *V. cholerae* classical biotype Type I-E CRISPR-Cas system | This study |
| KDS187 | Transductant in KS1265 background harboring PLE 1-KanR integrated in VCR in between VCA498 and VCA499. KS1265 added to supernatants of KDS3 infected with ICP1_2011_A ΔS9, KanR SpecR recipient selected and PLE insertion site confirmed by sequencing | This study |
| KDS188 | Transductant in KS1265 background harboring PLE 1-KanR integrated in VCR in between VCA491 and VCA492. KS1265 added to supernatants of KDS3 infected with ICP1_2011_A ΔS9, KanR SpecR recipient selected and PLE insertion site confirmed by sequencing | This study |
| KDS189 | Transductant in KS1265 background harboring PLE 1-KanR integrated in VCR in between VCA483 and VCA484. KS1265 added to supernatants of KDS3 infected with ICP1_2011_A ΔS9, KanR SpecR recipient selected and PLE insertion site confirmed by sequencing | This study |
| KDS190 | Transductant in KS1265 background harboring PLE 1-KanR integrated in VCR in between VCA440 and VCA441. KS1265 added to supernatants of KDS3 infected with ICP1_2011_A ΔS9, KanR SpecR recipient selected and PLE insertion site confirmed by sequencing | This study |
| KDS191 | Transductant in KS1265 background harboring PLE 1-KanR integrated in VCR in VCA354. KS1265 added to supernatants of KDS3 infected with ICP1_2011_A ΔS9, KanR SpecR recipient selected and PLE insertion site confirmed by sequencing | This study |
| KDS192 | Transductant in KS1265 background harboring PLE 1-KanR integrated in VCR in between VCA336 and VCA337. KS1265 added to supernatants of KDS3 infected with ICP1_2011_A ΔS9, KanR SpecR recipient selected and PLE insertion site confirmed by sequencing | This study |
| KDS193 | Transductant in KS1265 background harboring PLE 1-KanR integrated in VCR in between VCA312 and VCA313. KS1265 added to supernatants of KDS3 infected with ICP1_2011_A ΔS9, KanR SpecR recipient selected and PLE insertion site confirmed by sequencing | This study |
| KDS194 | Transductant in KS1265 background harboring PLE 1-KanR integrated in VCR in between VCA301 and VCA302. KS1265 added to supernatants of KDS3 infected with ICP1_2011_A ΔS9, KanR SpecR recipient selected and PLE insertion site confirmed by sequencing | This study |

**Supplementary Table S3: Plasmids used in this study**

| Plasmid | Description | Source |
| --- | --- | --- |
| pCRISPR | Derived from pMMB67EH, harbors *V. cholerae* CRISPR array under control of IPTG inducible promoter and insertion site for editing templates, AmpR | (7) |
| pTargetφCR1S1 -Ed ICP1 CRISPR array | pCRISPR cloned with anti-ICP1_2011_A S1 spacers and synthetic ICP1_2011_A CRISPR array with up and down arms of homology to permit recombination of synthetic array in phage genome. The synthetic phage CRISPR array harbors a BsaI site flanked by the phage direct repeats. Primers ACM485-512 with BsaI compatible arms were used to insert desired spacer sequences into this plasmid to render ICP1_2011_A with an engineered spacer | This study |
| pTargetφ*cas1*-EdCas1D244A | pCRISPR cloned with anti-*cas1* spacer directed against ICP1_2011_A, and an editing template to introduce D244A point mutation | This study |

**Supplementary Table S4: Primers used in this study**

| Primer use | Primer name | Sequence (5' to 3' orientation) | Source |
| --- | --- | --- | --- |
| HTM-PCR of Leader proximal spacer in CRISPR1 | KS395 | TCTCTAAATTTTTAATAAACCTTTGAAATTTATGGAT | This study |
|  | OLJ376 | GTGACTGGAGTTCAGACGTGTGCTCTTCCGATCTGGGGGGGGGGGGGGGG | (9) |
|  | KS397 | AATGATACGGCGACCACCGAGATCTACACTCTTTGAAATTTATGGATTTTGTGGTATAGTTTAAGTG | This study |
|  | BC43* | CAAGCAGAAGACGGCATACGAGATGTAGCCGTGACTGGAGTTCAGACGTGTGCTCTTCCGATCT | (10) |
|  | BC44* | CAAGCAGAAGACGGCATACGAGATTACAAGGTGACTGGAGTTCAGACGTGTGCTCTTCCGATCT | (10) |
|  | BC45* | CAAGCAGAAGACGGCATACGAGATTGTTGACTGTGACTGGAGTTCAGACGTGTGCTCTTCCGATCT | (10) |
| Illumina sequencing primer | KS396 | GGATTTTGTGGTATAGTTTAAGTGTTAGCAGCCGC | This study |
| q-PCR for PLE replication | Zac14 | AGGGTTTGAGTGCGATTACG | (8) |
|  | Zac15 | TGAGGTTTTACCACCTTTTGC | (8) |
| ICP1 gp58 detection | gp58F | AACGCTGCTTTTCCTTTTGA | (5) |
|  | gp58R | CCCAGCATTGAGGACACTTT | (5) |
| ICP1 CRISPR 1 detection | KS252 | CGGAGAAATTCAACAGTTATGG | (1) |
|  | KS253 | CAGAAAGTATTGCGGCTAGG | (1) |
| PLE detection | ACM349 | GGTGATTGTCCTTTCATTTGGG | This study |
|  | ACM350 | GTGATGCTGTTCAAACAGGG | This study |
|  | KS1034 | TGCATATCCATGCACAGGTG | This study |
|  | KS1035 | TGAGGTTTTACCACCTTTTGC | This study |
|  | KS1036 | GGAACAAACACAACAACGC | This study |
|  | KS1037 | TGTGTTGTTATGTTTGGTGC | This study |
|  | KS1038 | TTACATGTTGGAACTGGTCG | This study |
|  | KS1039 | CAGCGGCCAAAAATTCTTCC | This study |
| Construction of custom ICP1_2011_A CRISPR spacer array^a^ | ACM484 (-2.5 kb)** | CTAAC**AAGCCGAGAAAATAAAGGTGCTTGCTGGCATA** | This study |
|  | ACM485 (-2.5 kb)** | AAGA**TATGCCAGCAAGCACCTTTATTTTCTCGGCTT**G | This study |
|  | ACM486 (-1.5 kb)** | CTAAC**CAAAATTCTTCCTATAGTCTGCTGTATAACCA** | This study |
|  | ACM487 (-1.5 kb)** | AAGA**TGGTTATACAGCAGACTATAGGAAGAATTTTG**G | This study |
|  | ACM488 (22.5 kb)** | CTAAC**GTGGTTGTCCTTTCTTCTGGTTCAAGCAGCAT** | This study |
|  | ACM489 (22.5 kb)** | AAGA**ATGCTGCTTGAACCAGAAGAAAGGACAACCAC**G | This study |
|  | ACM490 (GG PAM)** | CTAAC**ATCCTGTTCGGTTCAATTGTCGAAGATGGTGA** | This study |
|  | ACM491 (GG PAM)** | AAGA**TCACCATCTTCGACAATTGAACCGAACAGGAT**G | This study |
|  | ACM492 (GT PAM)** | CTAAC**TCAACAGGTCGAGTTGATGAAAATTTTAGCTA** | This study |
|  | ACM493 (GT PAM)** | AAGA**TAGCTAAAATTTTCATCAACTCGACCTGTTGA**G | This study |
|  | ACM495 (-93 kb)** | CTAAC**ATCTTGAGAAACGAGTGTGGACAGTCCCCGAA** | This study |
|  | ACM496 (-93 kb)** | AAGA**TTCGGGGACTGTCCACACTCGTTTCTCAAGAT**G | This study |
|  | ACM497 (-71 kb)** | CTAAC**AGTCCCAATGAGCCGCGGTGGTTTCGGTTGTT** | This study |
|  | ACM498 (-71 kb)** | AAGA**AACAACCGAAACCACCGCGGCTCATTGGGACT**G | This study |
|  | ACM499 (-53 kb)** | CTAAC**ATTGAGCCGAGGTGGTTTCGGTTGTGGTGTTT** | This study |
|  | ACM500 (-53 kb)** | AAGA**AAACACCACAACCGAAACCACCTCGGCTCAAT**G | This study |
|  | ACM501 (-46 kb)** | CTAAC**ATTGAGCCGCAGTGGTTACGGTTATTGTGTTT** | This study |
|  | ACM502 (-46 kb)** | AAGA**AAACACAATAACCGTAACCACTGCGGCTCAAT**G | This study |
|  | ACM503 (-22 kb)** | CTAAC**CATATTGTCGCGAATGTCGAGGCTTGCATTGT** | This study |
|  | ACM504 (-22 kb)** | AAGA**ACAATGCAAGCCTCGACATTCGCGACAATATG**G | This study |
|  | ACM505 (0.5 kb)** | CTAAC**GAAGTTGGGATTTTGATGATTTTAGCCTTTAA** | This study |
|  | ACM506 (0.5 kb)** | AAGA**TTAAAGGCTAAAATCATCAAAATCCCAACTTC**G | This study |
|  | ACM509 (437 kb)** | CTAAC**TTTCGGTGGAGCAGTATCAAAATAGCGTTGTC** | This study |
|  | ACM510 (437 kb)** | AAGA**GACAACGCTATTTTGATACTGCTCCACCGAAA**G | This study |
| Deletion of mu-like region | KS802 | GGATCCCCAGCTTCGCGTCCCGCTACAAATAGTTTGGATCTCG | This study |
|  | KS803 | CGGTGAACGCTCTCCTGAGTTCACAAGGGAGTAGAAGCGG | This study |
|  | KS804 | ATGTTGCATCAGGGAACTGG | This study |
|  | KS805 | CAATGATAAAAGCAGCATGGG | This study |
| Deletion of mu2-like region | ACM103 | CACCGATGGAAATCTGTGG | This study |
|  | ACM104 | GGATCCCCAGCTTCGCGTCCGCCGACAATGTTAATGTAAGTGTG | This study |
|  | ACM105 | GCCTAACTATGGTGTGGTCG | This study |
| Construction of Cas1 D244A | ACM471*** | AAGAC**GTGGTGGGTTGGTTTTCGATGTAGCTGACATCA** | This study |
|  | ACM472*** | TTTC**TGATGTCAGCTACATCGAAAACCAACCCACCAC**G | This study |
|  | ACM478 | ATTATTGAAGTTTTGATGATAGCAGCTACATCGAAAACCAACCC | This study |
|  | ACM479 | TGCTATCATCAAAACTTCAATAATCTTACCTTTAGCTTTTCACGCTGC | This study |

| *The 6 bp barcode is underlined.  ^a^All oligos harbor BsaI compatible overhangs for insertion into pTargetφCR1S1 -Ed ICP1 CRISPR array. Target as in manuscript is in parentheses | | |
| --- | --- | --- |
| **The 32bp spacer sequence is in bold | | |
| ***The 33bp spacer sequence is in bold |  |  |

**Supplementary Table S5: The majority of spacers in the ICP1-related phages CRISPR**

**arrays show identity to *V. cholerae* PLEs**

| **Phage** | **CRISPR** | **Spacer** | **Sequence** | **Match (%)^a^** |
| --- | --- | --- | --- | --- |
| ICP1_2001_E | CR 1 | 1 | TGTGTCTATACTCAACCAATTTAAGCGCCGCA | ICP1 (81.25%) |
|  |  | 2 | CTACTCTCCCCAATATTAGCCATTCCTAATTCA | - |
|  |  | 3 | GTCACCTTACCGTAAGACAGGCAGTAAAATTA | PLE 2 (100%) |
|  |  | 4 | AAACTAGTGGACGTAATGCAGTATTCACGGTT | PLE 1 (100%) PLE 2 (90.62%) PLE 4 (100%) PLE 5 (100%) |
|  | CR 2 | 1 | ATCCACACTACAAATAGAACACTCAACCGTGA | PLE 1 (100%) PLE 2 (87.5%) PLE 3 (81.25%) PLE 4 (100%) PLE 5 (100%) |
|  |  | 2 | TATTGATTGGTCTCTAACCTTGGGATGATTAA | - |
|  |  | 3 | AGCGTGTGGGCTTTCATTTTTAAGCCAGTAAA | PLE 1 (90.62%) PLE 2 (90.62%) PLE 3 (90.62%) PLE 4 (87.5%) PLE 5 (100%) |
|  |  | 4 | TTCACGGGTAGCAACAGGGTAATAAACCAATA | PLE 1 (100%) PLE 2 (100%) PLE 4 (93.75%) PLE 5 (100%) |
| ICP1_2001_F | CR 1 | 1 | TGTGTCTATACTCAACCAATTTAAGCGCCGCA | ICP1 (81.25%) |
|  |  | 2 | CTACTCTCCCCAATATTAGCCATTCCTAATTCA | - |
|  |  | 3 | GTCACCTTACCGTAAGACAGGCAGTAAAATTA | PLE 2 (100%) |
|  |  | 4 | AAACTAGTGGACGTAATGCAGTATTCACGGTT | PLE 1 (100%) PLE 2 (90.62%) PLE 4 (100%) PLE 5 (100%) |
|  | CR 2 | 1 | ATCCACACTACAAATAGAACACTCAACCGTGA | PLE 1 (100%) PLE 2 (87.5%) PLE 3 (81.25%) PLE 4 (100%) PLE 5 (100%) |
|  |  | 2 | TATTGATTGGTCTCTAACCTTGGGATGATTAA | - |
|  |  | 3 | AGCGTGTGGGCTTTCATTTTTAAGCCAGTAAA | PLE 1 (90.62%) PLE 2 (90.62%) PLE 3 (90.62%) PLE 4 (87.5%) PLE 5 (100%) |
|  |  | 4 | TTCACGGGTAGCAACAGGGTAATAAACCAATA | PLE 1 (100%) PLE 2 (100%) PLE 4 (93.75%) PLE 5 (100%) |
| ICP1_2004_A | CR 1 | 1 | CATTGCAACTATGCAAAATGATGAAGCTAAAA | PLE 3 (100%) PLE 4 (100%) PLE 5 (100%) |
|  |  | 2 | TGTTAGAGTCGGTAGTATCTGGATGATCGATA | PLE 2 (100%) PLE 3 (100%) PLE 4 (100%) |
|  |  | 3 | TAGAAGAGTAATAGGAGCTACTGCAAACTTGT | - |
|  |  | 4 | TAACTATGTGTGGTTTATATTTTGTGTGCAAGA | - |
|  |  | 5 | TTTTGAAACTATTGACAGAAGGTTGGGAACCT | - |
|  |  | 6 | TTGAGGTTGAACCTCTTCCGGTTCCTCTTCTG | PLE 1 (93.75%) PLE 2 (100%) PLE 3 (84.38%) |
|  | CR 2 | 1 | GTGTATTGCTTGCAGTGGGTTACACACAAGAA | PLE 2 (84.28%) PLE 3 (87.5%) PLE 4 (90.62%) |
| ICP1_2005_A | CR 1 | 1 | TGTGTCTATACTCAACCAATTTAAGCGCCGCA | ICP1 (81.25%) |
|  |  | 2 | CTACTCTCCCCAATATTAGCCATTCCTAATTCA | - |
|  |  | 3 | AAACTAGTGGACGTAATGCAGTATTCACGGTT | PLE 1 (100%) PLE 2 (90.62%) PLE 4 (100%) PLE 5 (100%) |
|  |  | 4 | ATAATCGTTTTGAGTCTCACCAGCTTTTAGGC | PLE 2 (100%) PLE 3 (100%) |
|  | CR 2 | 1 | ATCCACACTACAAATAGAACACTCAACCGTGA | PLE 1 (100%) PLE 2 (87.5%) PLE 3 (81.25%) PLE 4 (100%) PLE 5 (100%) |
|  |  | 2 | TATTGATTGGTCTCTAACCTTGGGATGATTAA | - |
|  |  | 3 | TTCACGGGTAGCAACAGGGTAATAAACCAATA | PLE 1 (100%) PLE 2 (100%) PLE 4 (93.75%) PLE 5 (100%) |
| ICP1_2006_E | CR1 | 1 | TGTGTCTATACTCAACCAATTTAAGCGCCGCA | ICP1 (81.25%) |
|  |  | 2 | CTACTCTCCCCAATATTAGCCATTCCTAATTCA | - |
|  |  | 3 | GTCACCTTACCGTAAGACAGGCAGTAAAATTA | PLE 2 (100%) |
|  |  | 4 | AAACTAGTGGACGTAATGCAGTATTCACGGTT | PLE 1 (100%) PLE 2 (90.62%) PLE 4 (100%) PLE 5 (100%) |
|  | CR 2 | 1 | ATCCACACTACAAATAGAACACTCAACCGTGA | PLE 1 (100%) PLE 2 (87.5%) PLE 3 (81.25%) PLE 4 (100%) PLE 5 (100%) |
| ICP1_2009_A | CR 1 | 1 | CATTGCAACTATGCAAAATGATGAAGCTAAAA | PLE 3 (100%) PLE 4 (100%) PLE 5 (100%) |
|  |  | 2 | TGTTAGAGTCGGTAGTATCTGGATGATCGATA | PLE 2 (100%) PLE 3 (100%) PLE 4 (100%) |
|  |  | 3 | TCACAATCAGCTATAAGCCCTGCATTTTCAAT | PLE 2 (100%) |
|  |  | 4 | TTGTAGTGATGACATAATCTCGTCTCGACTCA | PLE 2 (100%) |
|  |  | 5 | AAGCAGAACTCACCGCCGAAGTGGAACAGCGT |  |
|  | CR 2 | 1 | GTGTATTGCTTGCAGTGGGTTACACACAAGAA | PLE 2 (84.28%) PLE 3 (87.5%) PLE 4 (90.62%) |
|  |  | 2 | AAGACGTGACAGCAGTGATCGACTTTATAACA | PLE 2 (100%) |
| ICP1_2011_A | CR 1 | 1 | CATTGCAACTATGCAAAATGATGAAGCTAAAA | PLE 3 (100%) PLE 4 (100%) PLE 5 (100%) |
|  |  | 2 | TGTTAGAGTCGGTAGTATCTGGATGATCGATA | PLE 2 (100%) PLE 3 (100%) PLE 4 (100%) |
|  |  | 3 | TTATGTATTGACCCCGACACGCCCCCCGACTG | - |
|  |  | 4 | TTACAGACGACCTAACTCTTCAGTACCATGAT | - |
|  |  | 5 | TACATAAGCTGCAACACGGTGTTCGTTTAAGT | PLE 2 (96.88%) PLE 4 (96.88%) |
|  |  | 6 | AAAATACGCCTTTTTCCCTTCATCGTTTAAAG | PLE 3 (90.62%) PLE 4 (100%) |
|  |  | 7 | ACCAACAAATCCCATAAACTGATAACCACGTT | - |
|  |  | 8 | GTCAACCCTTTGCTTATCTTCCCTATTTAAAT | PLE 1 (100%) PLE 2 (100%) |
|  |  | 9 | TGTTAACCACCGCTTGAAATAATCATGATGCA | PLE 1 (100%) PLE 2 (100%) |
| ICP1_2011_B | CR 1 | 1 | CATTGCAACTATGCAAAATGATGAAGCTAAAA | PLE 3 (100%) PLE 4 (100%) PLE 5 (100%) |
|  |  | 2 | TGTTAGAGTCGGTAGTATCTGGATGATCGATA | PLE 2 (100%) PLE 3 (100%) PLE 4 (100%) |
|  |  | 3 | TCACAATCAGCTATAAGCCCTGCATTTTCAAT | PLE 2 (100%) |
|  |  | 4 | TTGTAGTGATGACATAATCTCGTCTCGACTCA | PLE 2 (100%) |
|  |  | 5 | AAGCAGAACTCACCGCCGAAGTGGAACAGCGT | PLE 1 (100%) PLE 2 (100%) PLE 3 (84.38%) PLE 5 (81.25%) |
|  |  | 6 | TTGCATCAGTTGGATAGTTAATTGAGTGGGGC | PLE 1 (100%) PLE 4 (93.75%) PLE 5 (100%) |
|  | CR 2 | 1 | GTGTATTGCTTGCAGTGGGTTACACACAAGAA | PLE 2 (84.28%) PLE 3 (87.5%) PLE 4 (90.62%) |
|  |  | 2 | AAGACGTGACAGCAGTGATCGACTTTATAACA | PLE 2 (100%) |
| ICP1_2011_C | CR 1 | 1 | CATTGCAACTATGCAAAATGATGAAGCTAAAA | PLE 3 (100%) PLE 4 (100%) PLE 5 (100%) |
|  |  | 2 | TCACAATCAGCTATAAGCCCTGCATTTTCAAT | PLE 2 (100%) |
|  |  | 3 | TTGTAGTGATGACATAATCTCGTCTCGACTCA | PLE 2 (100%) |
|  |  | 4 | AAGCAGAACTCACCGCCGAAGTGGAACAGCGT | PLE 1 (100%) PLE 2 (100%) PLE 3 (84.38%) PLE 5 (81.25%) |
|  |  | 5 | TTGCATCAGTTGGATAGTTAATTGAGTGGGGC | PLE 1 (100%) PLE 4 (93.75%) PLE 5 (100%) |
|  | CR 2 | 1 | GTGTATTGCTTGCAGTGGGTTACACACAAGAA | PLE 2 (84.28%) PLE 3 (87.5%) PLE 4 (90.62%) |
|  |  | 2 | AAGACGTGACAGCAGTGATCGACTTTATAACA | PLE 2 (100%) |
| ICP1_2012_A | CR 1 | 1 | CATTGCAACTATGCAAAATGATGAAGCTAAAA | PLE 3 (100%) PLE 4 (100%) PLE 5 (100%) |
|  |  | 2 | TGTTAGAGTCGGTAGTATCTGGATGATCGATA | PLE 2 (100%) PLE 3 (100%) PLE 4 (100%) |
|  |  | 3 | TTTTGAAACTATTGACAGAAGGTTGGGAACCT | - |
|  |  | 4 | TTCAAAATCTTCCGATACATAACTAGCAAGTT | PLE 3 (100%) |
|  |  | 5 | TCACAATCAGCTATAAGCCCTGCATTTTCAAT | PLE 2 (100%) |
|  |  | 6 | TTGTAGTGATGACATAATCTCGTCTCGACTCA | PLE 2 (100%) |
|  |  | 7 | AATTGTCGAAGATGGTGAGGCACTAGCTACAC | PLE 1 (100%) PLE 2 (100%) |
|  |  | 8 | TGCGCAGCCACATCACAACACACTGTAAAAAT | PLE 1 (100%) PLE 4 (96.88%) PLE 5 (93.75%) |
|  |  | 9 | ACAAAACCTTAATAGGGACAAAAGTTATTAAA | PLE 1 (100%) PLE 3 (84.38%) PLE 4 (84.38%) PLE 5 (84.38%) |
|  | CR 2 | 1 | GTGTATTGCTTGCAGTGGGTTACACACAAGAA | PLE 2 (84.28%) PLE 3 (87.5%) PLE 4 (90.62%) |
|  |  | 2 | TTTTACGCAAAGTAGGATCGAGTGTTGCGAAC | *V. cholerae* (100%) |
| ICP1_2015_A | CR 1 | 1 | CATTGCAACTATGCAAAATGATGAAGCTAAAA | PLE 3 (100%) PLE 4 (100%) PLE 5 (100%) |
|  |  | 2 | TGTTAGAGTCGGTAGTATCTGGATGATCGATA | PLE 2 (100%) PLE 3 (100%) PLE 4 (100%) |
|  |  | 3 | TCACAATCAGCTATAAGCCCTGCATTTTCAAT | PLE 2 (100%) |
|  |  | 4 | TTGTAGTGATGACATAATCTCGTCTCGACTCA | PLE 2 (100%) |
|  |  | 5 | AAGCAGAACTCACCGCCGAAGTGGAACAGCGT | PLE 1 (100%) PLE 2 (100%) PLE 3 (84.38%) PLE 5 (81.25%) |
|  |  | 6 | TTTTGTTGTTATTTGTTATTTTGAATCAATCA | PLE 1 (100%) PLE 5 (100%) |
|  |  | 7 | TAGTTTCACCGTGCTATTATTCTCGACAACCA | PLE 1 (100%) PLE 2 (87.5%) |
|  | CR 2 | 1 | GTGTATTGCTTGCAGTGGGTTACACACAAGAA | PLE 2 (84.28%) PLE 3 (87.5%) PLE 4 (90.62%) |
|  |  | 2 | TTTTACGCAAAGTAGGATCGAGTGTTGCGAAC | *V. cholerae* (100%) |
| ICP1_2016_A | CR 1 | 1 | CATTGCAACTATGCAAAATGATGAAGCTAAAA | PLE 3 (100%) PLE 4 (100%) PLE 5 (100%) |
|  |  | 2 | TCACAATCAGCTATAAGCCCTGCATTTTCAAT | PLE 2 (100%) |
|  |  | 3 | TTGTAGTGATGACATAATCTCGTCTCGACTCA | PLE 2 (100%) |
|  |  | 4 | AAGCAGAACTCACCGCCGAAGTGGAACAGCGT | PLE 1 (100%) PLE 2 (100%) PLE 3 (84.38%) PLE 5 (81.25%) |
|  |  | 5 | TTGCATCAGTTGGATAGTTAATTGAGTGGGGC | PLE 1 (100%) PLE 4 (93.75%) PLE 5 (100%) |
|  | CR 2 | 1 | GTGTATTGCTTGCAGTGGGTTACACACAAGAA | PLE 2 (84.28%) PLE 3 (87.5%) PLE 4 (90.62%) |
|  |  | 2 | AAGACGTGACAGCAGTGATCGACTTTATAACA | PLE 2 (100%) |
| ICP1_2017_A | CR 1 | 1 | CATTGCAACTATGCAAAATGATGAAGCTAAAA | PLE 3 (100%) PLE 4 (100%) PLE 5 (100%) |
|  |  | 2 | TCACAATCAGCTATAAGCCCTGCATTTTCAAT | PLE 2 (100%) |
|  |  | 3 | AAGCAGAACTCACCGCCGAAGTGGAACAGCGT | PLE 1 (100%) PLE 2 (100%) PLE 3 (84.38%) PLE 5 (81.25%) |
|  |  | 4 | TTGCATCAGTTGGATAGTTAATTGAGTGGGGC | PLE 1 (100%) PLE 4 (93.75%) PLE 5 (100%) |
|  | CR 2 | 1 | GTGTATTGCTTGCAGTGGGTTACACACAAGAA | PLE 2 (84.28%) PLE 3 (87.5%) PLE 4 (90.62%) |
| ICP1_2017_B | CR 1 | 1 | CATTGCAACTATGCAAAATGATGAAGCTAAAA | PLE 3 (100%) PLE 4 (100%) PLE 5 (100%) |
|  |  | 2 | TCACAATCAGCTATAAGCCCTGCATTTTCAAT | PLE 2 (100%) |
|  |  | 3 | TTGCATCAGTTGGATAGTTAATTGAGTGGGGC | PLE 1 (100%) PLE 4 (93.75%) PLE 5 (100%) |
|  |  | 4 | TTCACGGGTAGCAACAGGGTAATAAACCAATA | PLE 1 (100%) PLE 2 (100%) PLE 4 (93.75%) PLE 5 (100%) |
|  | CR 2 | 1 | GTGTATTGCTTGCAGTGGGTTACACACAAGAA | PLE 2 (84.28%) PLE 3 (87.5%) PLE 4 (90.62%) |
|  |  | 2 | AAGACGTGACAGCAGTGATCGACTTTATAACA | PLE 2 (100%) |
| ICP1_2017_C | CR 1 | 1 | CATTGCAACTATGCAAAATGATGAAGCTAAAA | PLE 3 (100%) PLE 4 (100%) PLE 5 (100%) |
|  |  | 2 | TGTTAGAGTCGGTAGTATCTGGATGATCGATA | PLE 2 (100%) PLE 3 (100%) PLE 4 (100%) |
|  |  | 3 | TCACAATCAGCTATAAGCCCTGCATTTTCAAT | PLE 2 (100%) |
|  |  | 4 | TTGTAGTGATGACATAATCTCGTCTCGACTCA | PLE 2 (100%) |
|  |  | 5 | AAGCAGAACTCACCGCCGAAGTGGAACAGCGT | PLE 1 (100%) PLE 2 (100%) PLE 3 (84.38%) PLE 5 (81.25%) |
|  |  | 6 | TTGCATCAGTTGGATAGTTAATTGAGTGGGGC | PLE 1 (100%) PLE 4 (93.75%) PLE 5 (100%) |
|  | CR 2 | 1 | GTGTATTGCTTGCAGTGGGTTACACACAAGAA | PLE 2 (84.28%) PLE 3 (87.5%) PLE 4 (90.62%) |
|  |  | 2 | AAGACGTGACAGCAGTGATCGACTTTATAACA | PLE 2 (100%) |
| ICP1_2017_D | CR 1 | 1 | CATTGCAACTATGCAAAATGATGAAGCTAAAA | PLE 3 (100%) PLE 4 (100%) PLE 5 (100%) |
|  |  | 2 | TTGTAGTGATGACATAATCTCGTCTCGACTCA | PLE 2 (100%) |
|  |  | 3 | AAGCAGAACTCACCGCCGAAGTGGAACAGCGT | PLE 1 (100%) PLE 2 (100%) PLE 3 (84.38%) PLE 5 (81.25%) |
|  |  | 4 | TTGCATCAGTTGGATAGTTAATTGAGTGGGGC | PLE 1 (100%) PLE 4 (93.75%) PLE 5 (100%) |
|  | CR 2 | 1 | GTGTATTGCTTGCAGTGGGTTACACACAAGAA | PLE 2 (84.28%) PLE 3 (87.5%) PLE 4 (90.62%) |
|  |  | 2 | AAGACGTGACAGCAGTGATCGACTTTATAACA | PLE 2 (100%) |
| ICP1_2017_E | CR 1 | 1 | CATTGCAACTATGCAAAATGATGAAGCTAAAA | PLE 3 (100%) PLE 4 (100%) PLE 5 (100%) |
|  |  | 2 | TTGTAGTGATGACATAATCTCGTCTCGACTCA | PLE 2 (100%) |
|  |  | 3 | AAGCAGAACTCACCGCCGAAGTGGAACAGCGT | PLE 1 (100%) PLE 2 (100%) PLE 3 (84.38%) PLE 5 (81.25%) |
|  |  | 4 | TTGCATCAGTTGGATAGTTAATTGAGTGGGGC | PLE 1 (100%) PLE 4 (93.75%) PLE 5 (100%) |
|  | CR 2 | 1 | GTGTATTGCTTGCAGTGGGTTACACACAAGAA | PLE 2 (84.28%) PLE 3 (87.5%) PLE 4 (90.62%) |
|  |  | 2 | AAGACGTGACAGCAGTGATCGACTTTATAACA | PLE 2 (100%) |
| ICP1_2017_F | CR 1 | 1 | CATTGCAACTATGCAAAATGATGAAGCTAAAA | PLE 3 (100%) PLE 4 (100%) PLE 5 (100%) |
|  |  | 2 | TCACAATCAGCTATAAGCCCTGCATTTTCAAT | PLE 2 (100%) |
|  |  | 3 | AAGCAGAACTCACCGCCGAAGTGGAACAGCGT | PLE 1 (100%) PLE 2 (100%) PLE 3 (84.38%) PLE 5 (81.25%) |
|  |  | 4 | TTGCATCAGTTGGATAGTTAATTGAGTGGGGC | PLE 1 (100%) PLE 4 (93.75%) PLE 5 (100%) |
|  | CR 2 | 1 | GTGTATTGCTTGCAGTGGGTTACACACAAGAA | PLE 2 (84.28%) PLE 3 (87.5%) PLE 4 (90.62%) |
|  |  | 2 | AAGACGTGACAGCAGTGATCGACTTTATAACA | PLE 2 (100%) |

^a^ Spacers with no matches are indicated with a ‘-’.

**Supplementary Table S6: Number of spacers obtained in the high-throughput spacer acquisition assay and their respective targets**

|  | Replicate 1 | | Replicate 2 | | Replicate 3 | | Averages (%) | |
| --- | --- | --- | --- | --- | --- | --- | --- | --- |
| Target Region | Unique | All spacers | Unique | All spacers | Unique | All spacers | Unique | All spacers |
| PLE 1 | 1,760 | 1,113,858 | 1,697 | 1,408,934 | 1,625 | 1,245,537 | 1,694 (36.3) | 1,256,110 (96) |
| Chr I | 424 | 4,194 | 443 | 6,870 | 392 | 4,534 | 420 (9) | 5,199 (0.4) |
| Chr II | 2,623 | 32,981 | 2,562 | 60,360 | 2,487 | 47,634 | 2,557 (54.7) | 46,992 (3.6) |
| Total | 4,807 | 1,151,033 | 4,702 | 1,476,164 | 4,504 | 1,297,705 | 4,707 | 1,308,340 |
|  |  |  |  |  |  |  |  |  |
| Spacer Lengths | All spacers | | All spacers | | All spacers | | All spacers | |
| 31bp | 2,758 | | 3,711 | | 3,322 | | 3,264 (0.2) | |
| 32bp | 1,106,376 | | 1,413,306 | | 1,245,939 | | 1,255,207 (93) | |
| 33bp | 83,348 | | 101,746 | | 87,910 | | 91,001 (6.7) | |
| Total* | 1,192,482 | | 1,518,763 | | 1,337,171 | | 1,349,472 | |

***Not all putative spacers mapped to the targets.

**Supplementary Table S7: Number of additional putative engineered spacer binding sites on the *V. cholerae* small chromosome at decreasing levels of identity with the target.**

| **Engineered spacer (kb)** | **100% identity** | **90% identity** | **80% identity** |
| --- | --- | --- | --- |
| -93 | 1 | 0 | 0 |
| -71 | 1 | 94 | 14 |
| -53 | 1 | 28 | 72 |
| -46 | 1 | 39 | 64 |
| -22 | 1 | 0 | 0 |
| -2.5 | 1 | 0 | 0 |
| -1.5 | 1 | 0 | 0 |
| 0.5 | 1 | 1 | 2 |
| 22.5 | 1 | 0 | 0 |
| 437 | 1 | 0 | 0 |

**References:**

1. Seed KD, Lazinski DW, Calderwood SB, Camilli A. A bacteriophage encodes its own CRISPR/Cas adaptive response to evade host innate immunity. Nature. 2013;494(7438):489–91.

2. Crooks G, Hon G, Chandonia J, Brenner S. WebLogo: a sequence logo generator. Genome Res. 2004;14(6):1188–90.

3. Bawono P, Heringa J. PRALINE: A Versatile Multiple Sequence Alignment Toolkit. Methods Mol Bio. 2014;1079:245–62.

4. Naser I Bin, Hoque MM, Nahid MA, Rocky MK, Faruque SM. Analysis of the CRISPR-Cas system in bacteriophages active on epidemic strains of *Vibrio cholerae* in Bangladesh. Sci Rep. 2017;7(1):14880.

5. Seed KD, Bodi KL, Kropinski AM, Ackermann H-W, Calderwood SB, Qadri F, et al. Evidence of a Dominant Lineage of *Vibrio cholerae*-Specific Lytic Bacteriophages Shed by Cholera Patients over a 10-Year Period in Dhaka, Bangladesh. MBio. 2011;2(1):e00334-10.

6. Angermeyer A, Das MM, Singh DV, Seed KD. Analysis of 19 Highly Conserved *Vibrio cholerae* Bacteriophages Isolated from Environmental and Patient Sources Over a Twelve-Year Period. Viruses. 2018;10(6):299.

7. Box AM, McGuffie MJ, O’Hara BJ, Seed KD. Functional analysis of bacteriophage immunity through a Type I-E CRISPR-Cas system in *Vibrio cholerae* and its application in bacteriophage genome engineering. J Bacteriol. 2015;198(3):578–90.

8. O’Hara BJ, Barth ZK, McKitterick AC, Seed KD. A highly specific phage defense system is a conserved feature of the *Vibrio cholerae* mobilome. PLoS Genet. 2017;13(6):e1006838.

9. McDonough E, Lazinski DW, Camilli A. Identification of *in vivo* regulators of the *Vibrio cholerae xds* gene using a high-throughput genetic selection. Mol Microbiol. 2014;92(2):302–15.

10. Lazinski DW, Camilli A. Homopolymer tail-mediated ligation PCR: A streamlined and highly efficient method for DNA cloning and library construction. Biotechniques. 2013;54(1):25–34.
